# DENV-2 NS1 promotes AMPK-LKB1 interaction to activate AMPK/ERK/mTOR signaling pathway to induce autophagy

**DOI:** 10.1186/s12985-023-02166-0

**Published:** 2023-10-11

**Authors:** Ning Wu, Jinzhong Ji, Xiaoqin Gou, Pan Hu, Yao Cheng, Yuhang Liu, Yuanying Wang, Qilong Zhang, Li Zuo

**Affiliations:** 1 Chemistry and Biochemistry Laboratory, Guizhou Medical University, No.9, Beijing Road, Yunyan District, Guiyang, 550000 Guizhou Province China; 2 Department of Immunology, Guizhou Medical University, No.9, Beijing Road, Yunyan District, Guiyang, 550000 Guizhou Province China

**Keywords:** DENV-2, Autophagy, DENV-2 NS1, AMPK, LKB1, AMPK/ERK/mTOR signaling pathway

## Abstract

The global incidence of dengue fever has gradually increased in recent years, posing a serious threat to human health. In the absence of specific anti-dengue drugs, understanding the interaction of Dengue virus (DENV) with the host is essential for the development of effective therapeutic measures. Autophagy is often activated during DENV infection to promote viral replication, but the mechanism of how DENV's own proteins induce autophagy has not been clarified. In this study, we first preliminarily identified DENV-2 NS1 as the most likely viral protein for DENV-2-induced autophagy with the help of molecular docking techniques. Further experimental results confirmed that DENV-2 NS1 regulates DENV-2 infection of HUVEC-induced autophagy through the AMPK/ERK/mTOR signaling pathway. Mechanistically, DENV-2 NS1 mainly interacted with AMPK by means of its Wing structural domain, and NS1 bound to all three structural domains on the AMPKα subunit. Finally, the experimental results showed that DENV-2 NS1 promoted the interaction between LKB1 and AMPKα1 and thus activated AMPK by both increasing the expression of LKB1 and binding LKB1. In conclusion, the results of this study revealed that DENV-2 NS1 protein served as a platform for the interaction between AMPK and LKB1 after DENV-2 infection with HUVEC, and pulled AMPK and LKB1 together to form a complex. LKB1 to form a complex, promoting LKB1 action on the kinase structural domain of AMPKα1, which in turn promotes phosphorylation of the Thr172 site on the AMPK kinase structural domain and activates AMPK, thereby positively regulating the AMPK/ERK/mTOR signaling pathway and inducing autophagy. The present discovery improves our understanding of DENV-2-induced host autophagy and contributes to the development of anti-dengue drugs.

**Supplementary Information:**

The online version contains supplementary material available at 10.1186/s12985-023-02166-0.

## Introduction

In recent decades, the number of DENV cases has gradually increased, with an estimated 300–400 million people being infected each year, and each outbreak causing severe disease and economic burden [1, 2]. There is no specific drug for dengue fever, and the dengue vaccine may reduce the incidence of dengue fever, although concerns about its overall efficacy and safety remain [3]. Therefore, an in-depth understanding of the pathogenesis of the virus facilitates the development of effective therapeutic drugs and preventive interventions.

DENV infection triggers an innate immune response to produce antiviral effects in the body, with progressive evolution, the virus may even exacerbate the disease by evading recognition or inhibiting the production of antiviral states [4]. In particular, autophagy has been shown to be activated in DENV-infected cells, and most studies suggest that DENV-2-induced autophagy promotes viral self-RNA replication, while inhibition of autophagy leads to a significant decrease in viral replication [5, 6]. Early DENV-induced autophagy proteins ATG5-ATG12 are involved in suppressing MAVS-mediated ISG induction, thereby promoting viral replication [7]. Meanwhile, the release of infectious autophagy-associated dengue vesicles from DENV-infected cells can protect viral RNA within the vesicles and avoid being neutralized by antibodies that could facilitate virus transmission [8]. In terms of energy metabolism, DENV infection-induced autophagy increases cellular glucose uptake, transport and glycolysis, thereby promoting viral replication [9]. It also promotes lipolysis to increase the production of ATP to enhance viral replication [10].

Activated AMPK inhibits mTORC1 activity, which activates autophagy and induces lipophagy, enhancing the breakdown of lipid droplets (LD), especially triglycerides, to promote ATP production to enhance viral replication. In addition, activated AMPK can also promote autophagy in an mTOR-independent manner by directly phosphorylating ULK1/2 and phosphatidylinositol-3-kinase (VPS34-Beclin complex) [11]. The previous study of our team found that DENV-2 induces autophagy in HUVECs through the AMPK/ERK/mTOR signaling pathway [12, 13]. However, the viral components and molecular mechanisms behind DENV-2 regulation of the AMPK/ERK/mTOR signaling pathway to induce autophagy are not yet clear.

DENV is an RNA virus with a genome size of approximately 11 kb, encoding three structural proteins (E, prM and C) and seven non-structural proteins (NS1, NS2A, NS2B, NS3, NS4A, NS4B and NS5) [14]. Structural proteins are mainly involved in viral RNA capsidization, while non-structural proteins are mainly involved in viral replication [15]. NS1 is important for viral replication and infectious particle production, and upon secretion is involved in viral immune evasion; in infected cells, NS1 also binds to the luminal side of the endoplasmic reticulum vesicle membrane and helps anchor the viral replication complex [16]. Although the functional studies of DENV nonstructural proteins have been relatively abundant, the viral proteins involved in DENV-induced autophagy have been less reported. Specifically, only NS1 and NS4A have been reported to have an autophagy-inducing role during DENV infection. However, to date, there are no reports on DENV's own proteins regulating autophagy by modulating the AMPK pathway.

In the present study, we uncovered a novel mechanism by which NS1 mediates DENV-2-induced autophagy. Our data suggest that DENV-2 NS1 acts as an assembly platform to promote AMPK-LKB1 interaction to activate AMPK, and interestingly NS1 not only promotes AMPK and LKB1 binding, but also promotes LKB1 expression. A more specific mechanism is that DENV-2 NS1 protein promotes the binding of AMPK upstream kinase LKB1 to the kinase structural domain of AMPKa1 subunit mainly by its Wing domain, which in turn promotes the phosphorylation of Thr172 site on AMPK kinase structural domain to activate AMPK, thus positively regulating AMPK/ERK/mTOR signaling pathway and inducing autophagy.

## Results

### NS1 among the non-structural proteins of DENV-2 is most likely to be involved in regulating the AMPK/ERK/mTOR pathway to induce autophagy

**Fig. 1:**
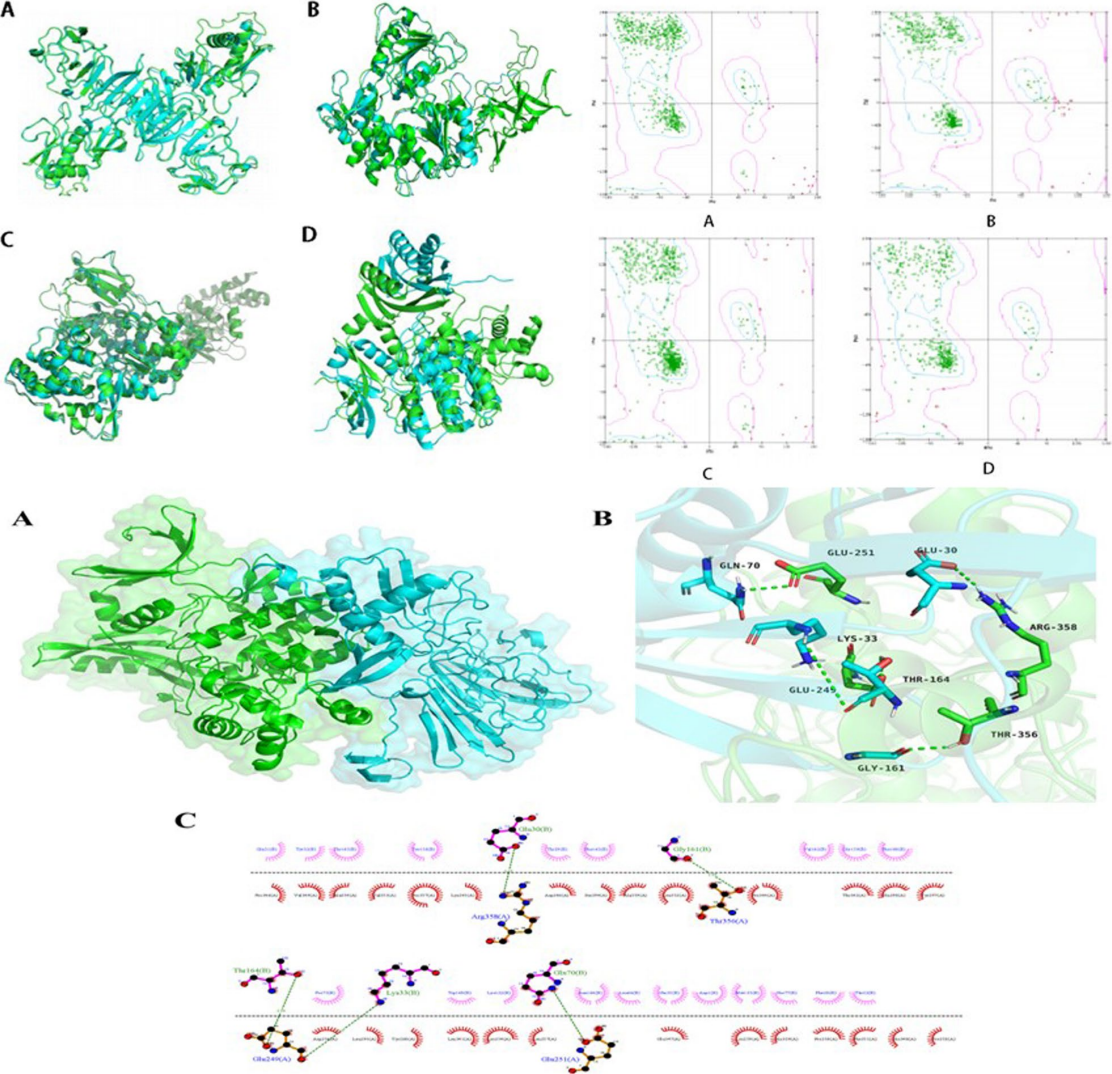
NS1 is most likely involved in regulating the AMPK/ERK/mTOR pathway to induce autophagy. **A** Superposition of protein homology modeling structure and natural structure. Green is the homology modeled structure, blue is the natural structure, and from a–d, the superposition of NS1, NS3, NS5, and AMPKα homology modeled and natural structures in order. **B** Pull-down diagram of the protein structure. From a–d, NS1, NS3, NS5, and AMPKα in order. **C** Conformation of NS1 complexed with AMPK α1. Where NS1 protein is in blue and AMPK1 protein is in green. a is the overall binding mode of the complex, b is the protein–protein interaction amino acid residues, and c is the 2D diagram of residue interactions

To initially determine which non-structural proteins of dengue virus may be involved in AMPK pathway-mediated autophagy, we used molecular docking techniques to predict the affinity of the major non-structural proteins of dengue virus to AMPK proteins. Firstly, the protein structures of NS1, NS3, NS5 and AMPKα were constructed by homology modeling, and the RMSD values of NS1_RMSD = 0.784, NS3_RMSD = 1.055, NS5_RMSD = 0.953 and AMPKα_RMSD = 8.247 were calculated for each target protein respectively. The stacking situation was relatively good, and the stacking situation is shown in Fig. 1A. Besides, we also use stereochemical evaluation to mainly check whether the homologous modeling model conforms to the conventional parameters such as bond lengths, bond angles, dihedral angles, etc., and the results are generally presented by means of a Rasch plot. More than 90% of the amino acid residues of the homology modeled protein structure model were in the best rational and permissive regions, indicating the high quality and reliability of the modeled protein structure, and the pull-down diagram of each protein is presented in Fig. 1B. Finally, following two rounds of calculations of ZDOCK and RDOCK, we selected the lowest scoring in Pose as the main complex conformation. The docking calculation scoring results indicated that the AMPKα protein had the best docking score with NS1 (Table 1), suggesting that NS1, the main nonstructural protein of DENV-2, has the highest potential to interact with AMPK. Figure 1C illustrates the conformation of NS1 complexed with AMPKα1. Further comparison of the proteins on the AMPK/ERK/mTOR pathway with the NS1 docking score shows that AMPK has the greatest potential to interact with NS1 (Table 1). This suggests that NS1 may interact with AMPK and thus regulate the AMPK/ERK/mTOR signaling pathway, which in turn mediates the autophagy induced during DENV-2 infection.

**Table 1:** Protein docking score

Protein complex	ZDock score	E_RDock
NS1&AMPK α	23.12	− 13.4844
NS3&AMPK α	22.30	− 8.38646
NS5&AMPK α	23.60	− 7.19943
NS1&ERK	23.50	2.42205
NS1&mTOR	27.64	2.44442

### NS1 activates autophagy through the AMPK/ERK/mTOR signaling pathway

**Fig. 2:**
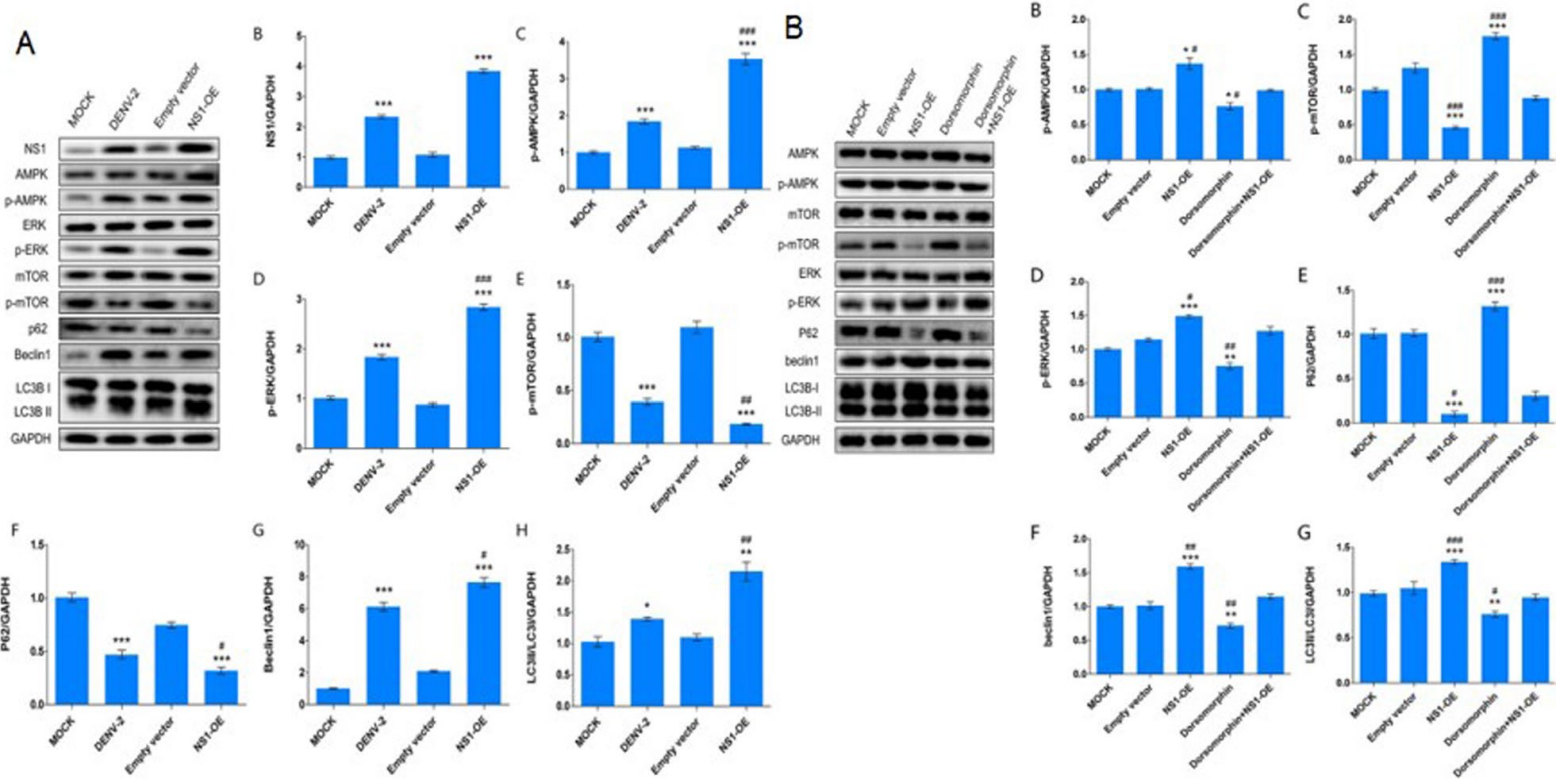
DENV-2 NS1 induces autophagy through AMPK/ERK/mTOR signaling pathway. **A** Protein immunoblotting was used to detect changes in the AMPK/ERK/mTOR signaling pathway and autophagy-related molecules in four groups of MOCK, DENV-2, Empty vector and NS1-OE. **B** Protein immunoblotting was used to detect changes in AMPK/ERK/mTOR signaling pathway and autophagy-related molecules in five groups of MOCK, Empty vector and NS1-OE, Dorsomorphin, Dorsomorphin + NS1-OE. *represents comparison with MOCK, *p < 0.05, **p < 0.01, ***p < 0.001; # represents comparison with Dorsomorphin + DENV-2 NS1-OE group, #*p* < 0.05, ##*p* < 0.01, ##*p* < 0.001

To further verify the effect of NS1, the effect of DENV-2 NS1 on AMPK pathway and autophagy was examined after overexpression of NS1. The Western blotting results demonstrated that both AMPK and ERK activation levels were effectively increased and mTOR activation levels were greatly reduced after increased NS1 expression. In addition, P62 expression was reduced, and Beclin1 expression and LC3II/LC3I expression were both substantially increased (Fig. 2A). Meanwhile, the NS1-OE group had higher levels of AMPK/ERK/mTOR pathway activation and more pronounced autophagy activation compared with the DENV-2 group (Fig. 2A). These findings suggest that NS1 may mediate the effect of activation of AMPK/ERK/mTOR pathway by DENV-2 to induce autophagy.

To confirm further that NS1-induced autophagy was mediated by the AMPK/ERK/mTOR signaling pathway, we added an AMPK inhibitor (Dorsomorphin) to cells overexpressing NS1 and examined the activation level of AMPK/ERK/mTOR signaling pathway and the degree of activation of autophagy. Compared with the NS1-OE group, the NS1-OE + Dorsomorphin group showed decreased levels of AMPK and ERK phosphorylation, increased levels of mTOR phosphorylation, increased autophagy-related protein P62, decreased Beclin1, and decreased LC3II/LC3I (Fig. 2B). Together, this indicated that the effect of NS1 to promote activation of AMPK/ERK/mTOR signaling pathway to induce autophagy could be reversed by AMPK inhibitors, suggesting that NS1 induces autophagy via AMPK/ERK/mTOR signaling pathway.

### DENV-2 NS1 interacts with AMPK

**Fig. 3:**
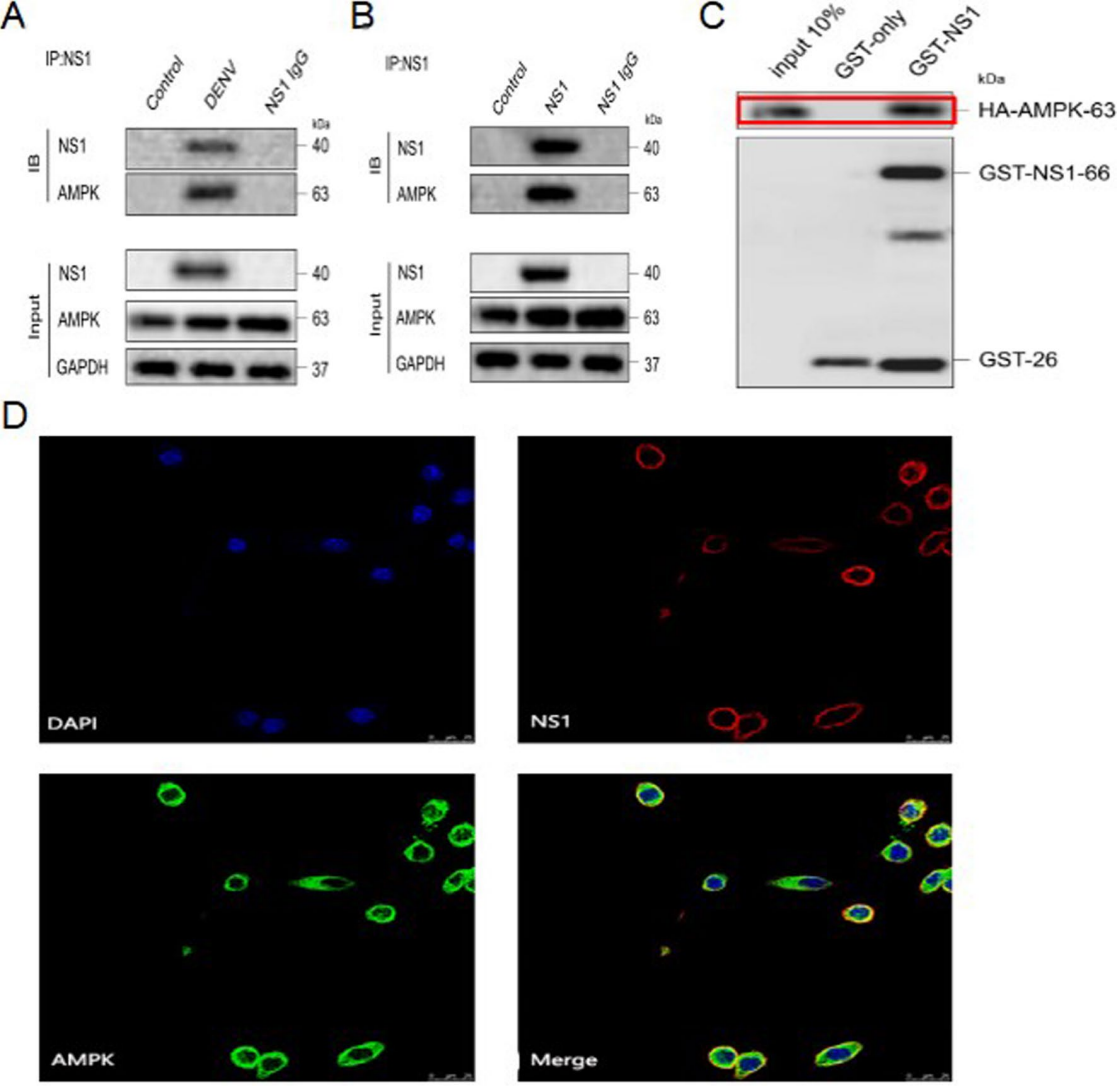
DENV2 NS1 interacts with AMPK. **A** Interaction of NS1 with AMPK in DENV-2 infected HUVEC. **B** Interaction between NS1 and AMPK in HUVEC overexpressing NS1. **C** Direct interaction between NS1 and AMPK. (**D**) Co-localization of NS1 and AMPK exists in HUVEC cells overexpressing NS1. Red fluorescence indicates DENV-2 NS1, blue fluorescence indicates nucleus, and green fluorescence indicates AMPK. scale bar: 25 μm

Immunoprecipitation (Co-IP) assay was performed to detect whether DENV-2 and AMPK interacted with each other. The experimental results showed that either DENV-2 NS1 or AMPK could be precipitated upon DENV-2 infection or NS1 overexpression (Fig. 3A, B). Moreover, the results of GST pulldown assay demonstrated that GST-NS1 binds to HA-AMPK compared to GST-only (Fig. 3C). It was confirmed that DENV2 NS1 and AMPK have a direct binding effect.

Laser confocal microscopy results exhibited that DENV-2 NS1 protein was localized in the cytoplasm and AMPK protein was also localized in the cytoplasm of DENV-2-infected HUVECS for 48 h. The co-localization of the two proteins was evident (Fig. 3D). All of above data revealed that DENV-2 NS1 protein and AMPK protein interacted and co-localized in DENV-2-infected HUVECS.

### DENV-2 NS1 wing structural domain acts critically in the interaction between NS1 and AMPK

**Fig. 4:**
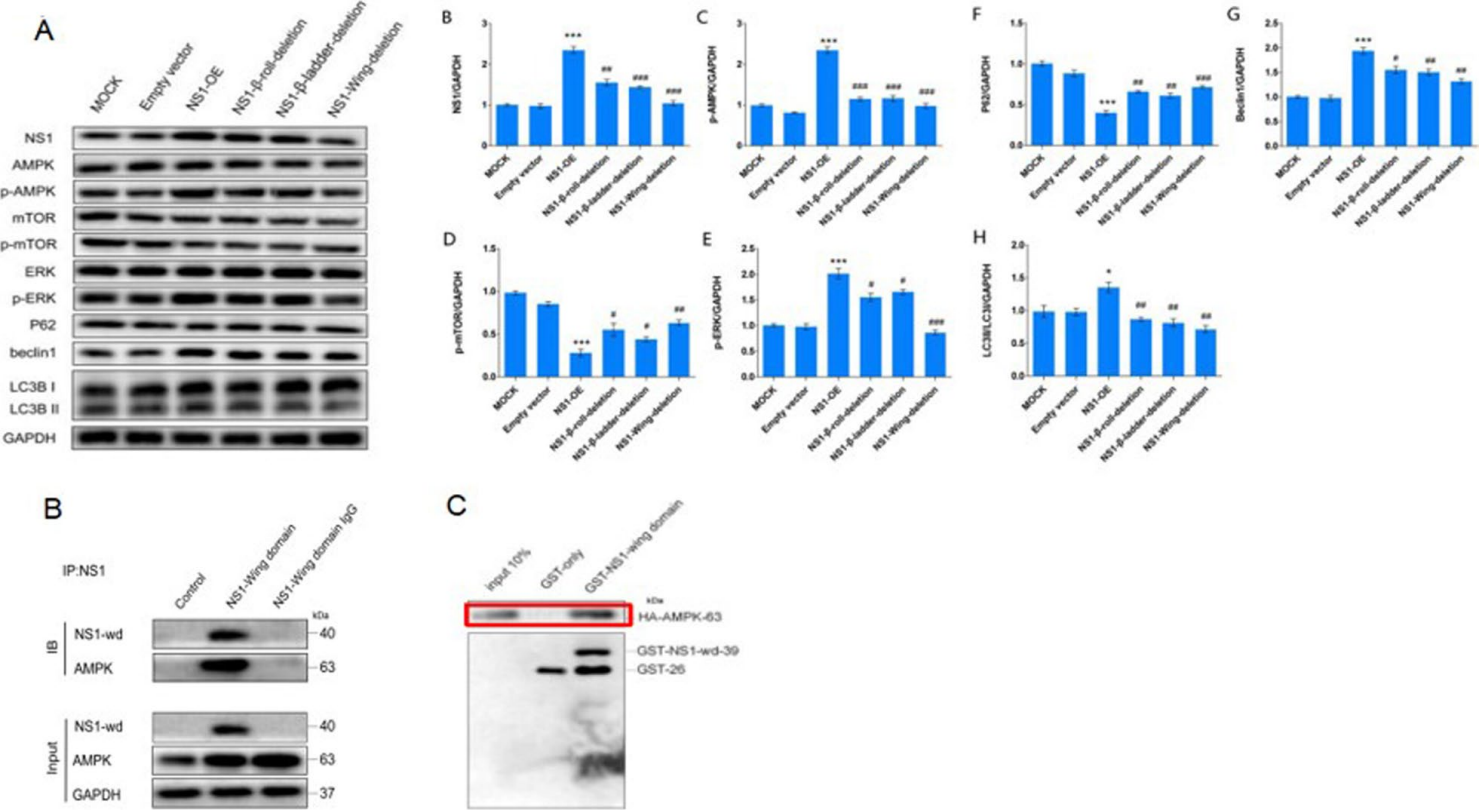
The DENV-2 NS1 Wing domain plays a key role in the interaction of NS1 with AMPK. **A** Westernblots detection of changes in related molecules upon deletion of different structural domains of NS1. * represents comparison with MOCK, *p < 0.05, **p < 0.01, ***p < 0.001; # represents comparison with DENV-2 NS1-OE group, #*p* < 0.05, ##*p* < 0.01, ##*p* < 0.001. **B** Co-ip. **C** The fusion proteins DENV-2 NS1-Wing domain with GST tag, GST-only and AMPK with HA tag were subjected to GST pulldown assay in vitro, respectively

Three NS1 structural domain deletions were constructed and transfected with HUVECs. western blots experiments showed that all three structural domain deletion groups could weaken the effect of NS1 to different degrees, among which the NS1-wing-deletion group had the most significant weakening of NS1 effect. This partial result suggests that all three structural domains are essential for NS1 function, and the NS1-wing structural domain may be a key structural domain for NS1 activation of autophagy. The interaction between DENV-2 NS1-Wing and AMPK was further analyzed by Co-IP. The results showed that the NS1-Wing domain was precipitated, along with AMPK (Fig. 4B). This indicates that there is an interaction between the two. Similarly, the results of GST pulldown experiments showed that GST-NS-wd could bind to HA-AMPK compared with GST-only (Fig. 4C), confirming a direct interaction between NS1-Wing domain and AMPK.

### NS1 interacts with the S, C, and U structural domains of AMPKa

**Fig. 5:**
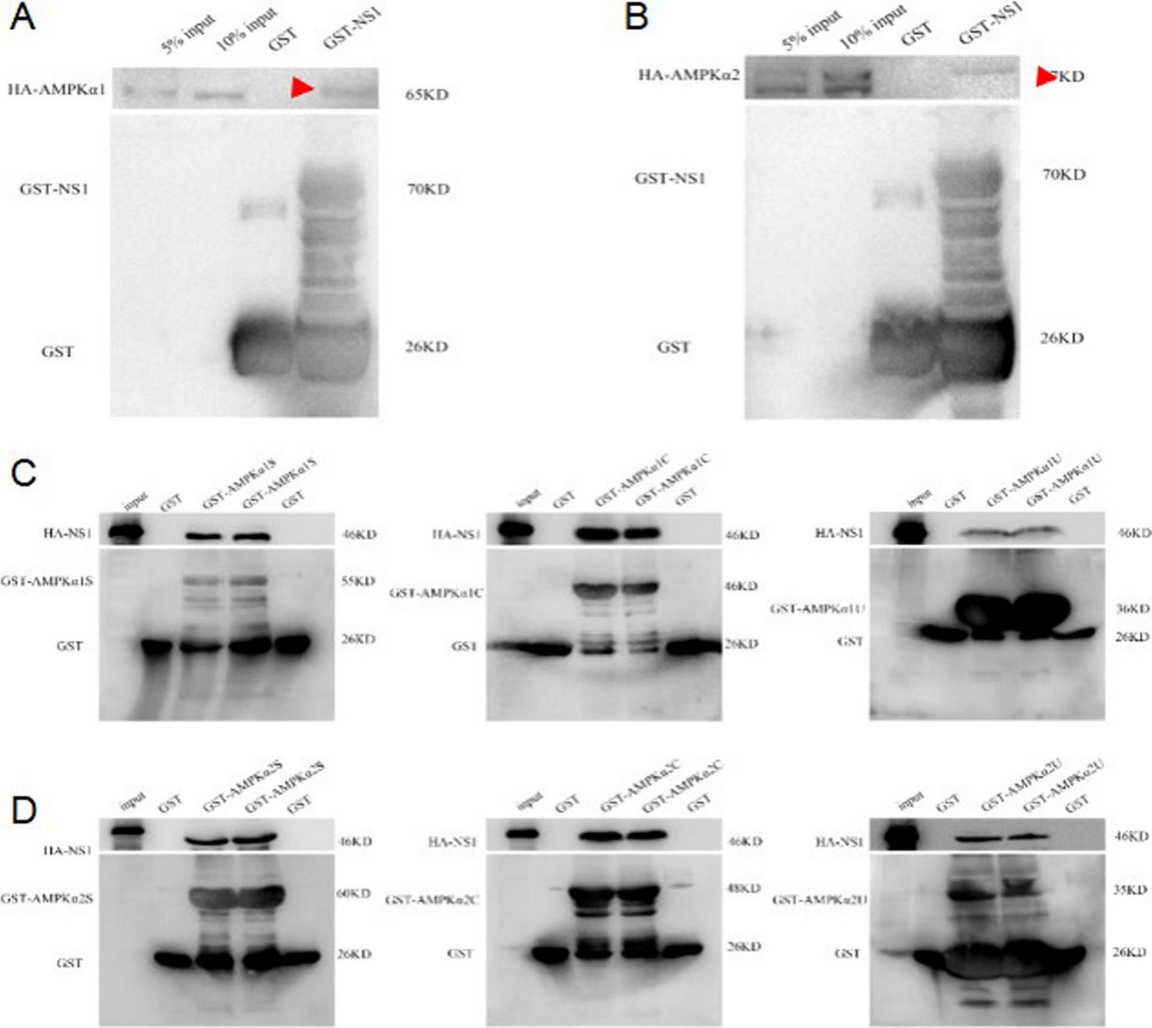
Interaction of DENV NS1 with the S, C, and U structural domains of AMPKa. **A** The fusion proteins DENV-2 NS1 and GST-only with GST tag and AMPKα1 with HA tag were subjected to GST pulldown assay in vitro, respectively. **B** The fusion proteins DENV-2 NS1 and GST-only with GST tag and AMPKα2 with HA tag were subjected to GST pulldown assay in vitro, respectively. **C** AMPKα1 was divided into three structural domains by STC, C-terminal, and UBA-like, and glutathione gel beads loaded with GST protein and GST-AMPKα1S, GST-AMPKα1C, and GST-AMPKα1U proteins were co-incubated with HA-tagged NS1 expressed in vitro, respectively. **D** AMPKα2 was divided into STC, C-terminal, and UBA-like structural domains by structural domain, and glutathione gel beads loaded with GST protein and GST-AMPKα2S, GST-AMPKα2C, and GST-AMPKα2U proteins were co-incubated with HA-tagged NS1 expressed in vitro, respectively

Subsequently, we explored which structural domain of AMPK plays a key role in the interaction between DENV2 NS1 and AMPK. First, the results of GST pulldown experiments indicated that GST-NS1 could bind to HA-AMPKα1 and AMPKα2 (Fig. 5A, B), confirming a direct binding interaction between DENV2 NS1 and AMPKα. Then, we divided AMPKα1 and AMPKα2 into three structural domains, STC (containing the kinase structural domain), C-terminal (containing the C-terminal structural domain), and UBA-like (containing the self-inhibitory structural domain) by structural domains, and then performed GST pulldown experiments. The outcomes suggested that NS1 bound to all three fragments of AMPKα, implying that the promotion of AMPK phosphorylation by NS1 might be mediated by multiple mechanisms, but the binding of the STC and C-terminal structural domains of AMPKα to NS1 was stronger, while the binding of UBA-like to NS1 was weaker (Fig. 5C, D). It indicates that the kinase structural domains STC and C-terminal structural domains of AMPKα may be the main structural domains of NS1 interacting with AMPK, and the promotion of AMPK phosphorylation by NS1 may be mediated via these structural domains.

### LKB1 participates in the activation of AMPK by NS1

**Fig. 6:**
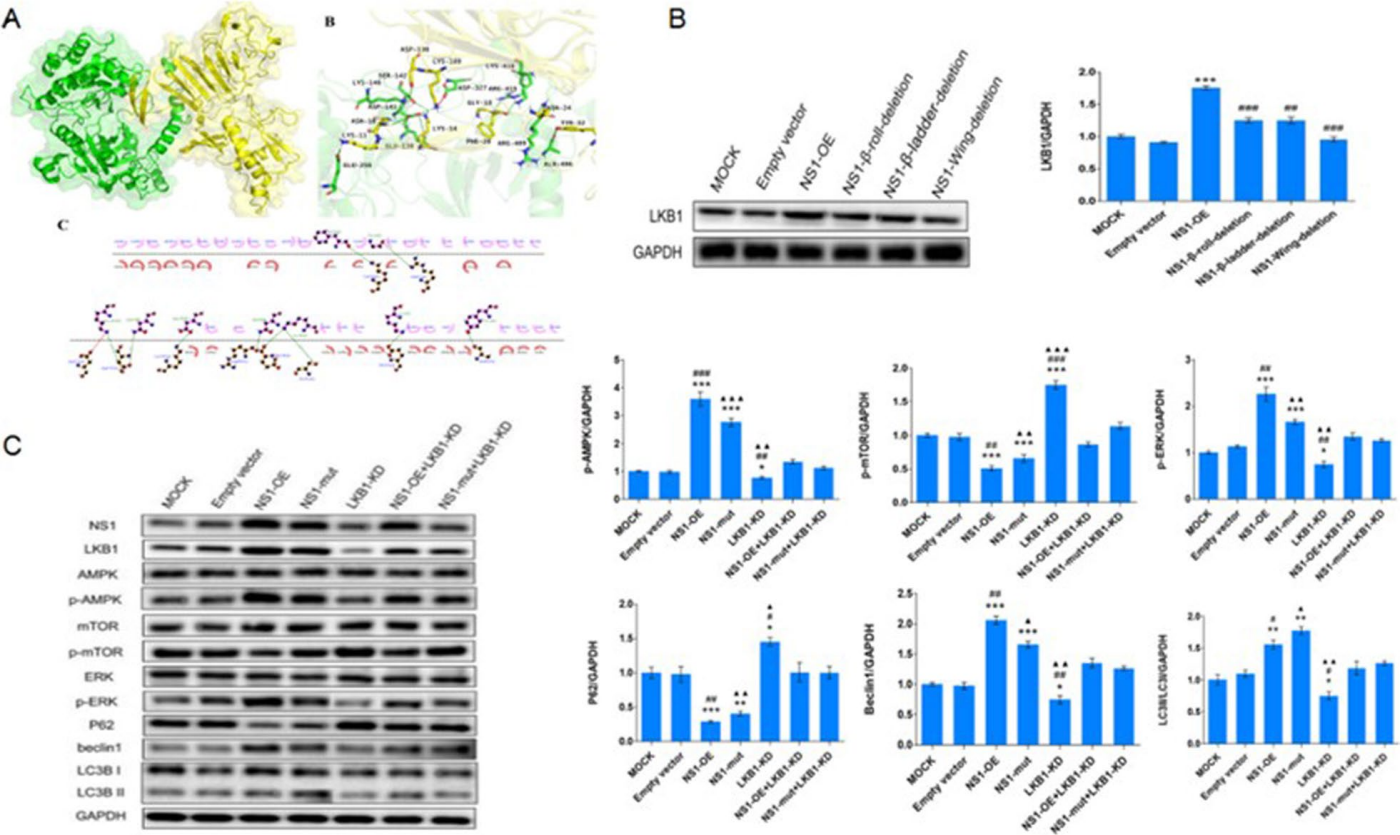
LKB1 is involved in the activation of AMPK by NS1. **A** Conformation of the NS1-LKB1 complex. NS1 protein is shown in blue and AMPK1 protein is shown in green. a is the overall binding mode of the complex, b is the protein–protein interaction amino acid residues, and c is the 2D map of residue interactions. **B** Effect of NS1 and its structural domain on LKB1 expression. **C** LKB1 knockdown attenuates the effect of NS1-induced autophagy. * represents comparison with MOCK, *p < 0.05, **p < 0.01, ***p < 0.001; # represents comparison with DENV-2 NS1-OE + LKB1-KD group, #*p* < 0.05, ##*p* < 0.01, ##*p* < 0.001; △ represents comparison with DENV-2 NS1-mut + LKB1-KD group, △*p* < 0.05, △△*p* < 0.01, △△△*p* < 0.001

The docking calculation scoring suggested that the ERDock score of NS1 with LKB1 was lower (see Table 2). It indicates that the interaction of NS1 is more likely between NS1 protein and LKB1, and LKB1 may be involved in the activation of AMPK by NS1. The conformation of NS1 complex with LKB1 is shown in Fig. 6A.

**Table 2:** Protein docking score

Protein complex	ZDock score	E_RDock
NS1&LKB1	23.20	− 26.1365
NS1&CAMKK2	22.06	− 8.66241

Westernblots results displayed that the LKB1 expression level in the NS1-OE group was markedly higher than that in the NC group. Compared with the NS1-OE group, LKB1 levels were reduced in the NS1 deletion group, with the NS1-wing-deletion group being more pronounced (Fig. 6B), indicating that NS1 can promote LKB1 expression.

Next, the effect of knockdown of LKB1 on the activation of AMPK/ERK/mTOR signaling pathway by NS1 to induce autophagic effect was verified. The results revealed that AMPK and ERK phosphorylation levels, Beclin1 expression, LC3II/LC3I were clearly decreased and mTOR phosphorylation levels and P62 expression were increased in the NS1-OE + LKB1-KD group compared to the NS1-OE group (Fig. 6C). This indicates that the knockdown of LKB1 reversed the effect of NS1, and the effect of NS1 activation of AMPK/ERK/mTOR signaling pathway to induce autophagy required LKB1 mediation. And the effect of NS1-Wing domain also requires the mediation of LKB1.

### NS1 acts as the assembly platform for LKB1-AMPK interactions and promotes LKB1-AMPK interactions

**Fig. 7:**
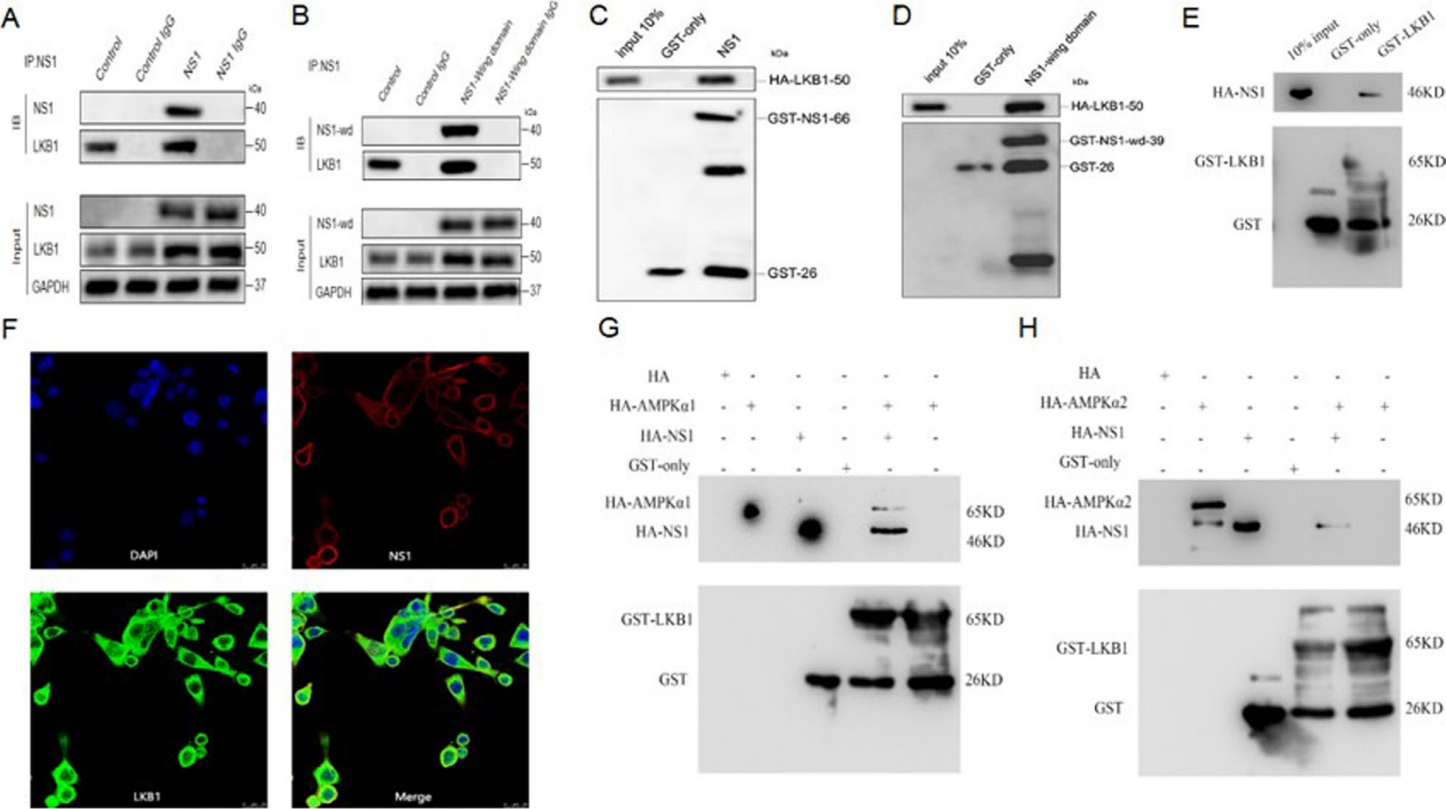
NS1 acts as an assembly platform for LKB1-AMPK interactions and promotes LKB1-AMPK interactions. **A** NS1 interacts with LKB1. **B** NS1-Wing domain interacts with LKB1. **C** NS1 interacts directly with LKB1. **D** NS1-Wing domain has a direct interaction with LKB1. **E** Direct interaction between LKB1 and NS1. **F** Co-localization of NS1 with LKB1 exists in HUVEC cells overexpressing NS1. Red fluorescence indicates DENV-2 NS1, green fluorescence indicates LKB1, and blue fluorescence indicates nuclei. Scale bar: 25 μm. **G** NS1 promotes the interaction of AMPKα1 with LKB1. Glutathione gel beads loaded with GST protein and GST-LKB1 protein were co-incubated with in vitro-expressed HA-tagged DENV-2 NS1 and AMPKα1 in the presence and absence of DENV-2 NS1 addition, respectively. **H** Effect of NS1 on the interaction of AMPKα2 with LKB1. Glutathione gel beads loaded with GST protein and GST-LKB1 protein were co-incubated with in vitro-expressed HA-tagged DENV-2 NS1 and AMPKα2, respectively, with and without DENV-2 NS1 addition

To further dissect the intrinsic relationship between NS1 and AMPK and LKB1, we examined whether NS1 and its Wing domain interact with LKB1 with the help of CO-IP and GST pulldown assays. Co-IP findings exhibited that after the precipitation reaction using NS1 antibody, NS1 was precipitated and LKB1 could also be precipitated (Fig. 7A), indicating an interaction between the two. Similarly, the NS1-Wing domain also interacted with LKB1 (Fig. 7B). GST pulldown assays displayed that GST-NS1 and GST-NS1-Wing domain binds to HA-LKB1 compared to GST-only (Fig. 7C, D), confirming a direct binding interaction between DENV2 NS1 and AMPK. In addition, the GST-tagged fusion proteins LKB1 and GST-only were subjected to GST pulldown assays in vitro with HA-tagged NS1, respectively, and GST-LKB1 was able to bind to HA-NS1 compared with GST-only (Fig. 7E), indicating that both NS1 and its Wing domain have direct interaction with LKB1. Laser confocal microscopy revealed that LKB1 protein was mainly localized in the cytoplasm with a little localization in the nucleus, while NS1 protein was localized in the cytoplasm, and a clear co-localization could occur between the two (Fig. 7F). This implies that LKB1 is co-localized with NS1 in the cytoplasm.

We hypothesized that NS1 might activate AMPK by promoting LKB1 expression and facilitating its binding to AMPK. to further prove our conjecture, we co-incubated GST and GST-LKB1 with HA-tagged NS1 and AMPKα, respectively. It was found that there was no interaction between AMPKα1, AMPKα2 and LKB1 without the addition of NS1, and when HA-NS1 was added to the reaction system, there was interaction between AMPKα1 and LKB1, but no interaction between AMPKα2 and LKB1 (Fig. 7G). The above results indicated that NS1 specifically promoted the binding of LKB1 and AMPKα1.

## Discussion

**Fig. 8:**
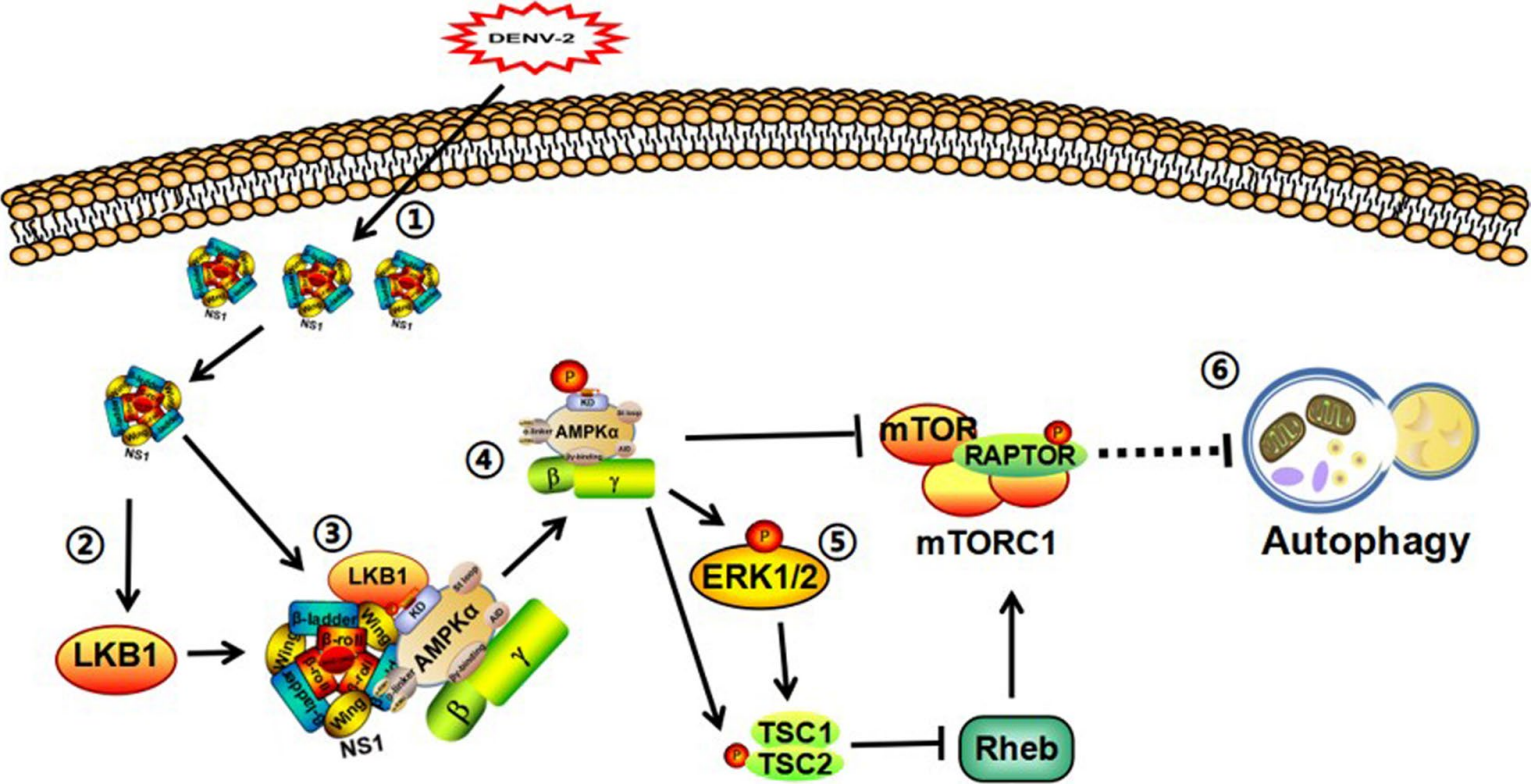
DENV-2 NS1 promotes AMPK-LKB1 interaction to activate AMPK/ERK/mTOR signaling pathway to induce autophagy

In the present study, we found that DENV-2 NS1 protein promoted the binding of AMPK upstream kinase LKB1 to the kinase structural domain of AMPKa1 mainly through its Wing structural domain after DENV-2 infection of HUVEC. Furthermore, it promotes phosphorylation of the Thr-172 site on the AMPK kinase structural domain and activates AMPK, which positively regulates the AMPK/ERK/mTOR signaling pathway and induces autophagy (Fig. 8).

DENV causes dengue hemorrhagic fever (DHF) characterized by vascular leakage and coagulation disorders, among others. Vascular leakage leads to hemoconcentration and plasma fluid accumulation, resulting in circulatory collapse and further development of life-threatening dengue shock syndrome (DSS) [17]. NS1 is the only protein continuously secreted by infected host cells, which is synthesized as a monomer and processed in the endoplasmic reticulum and trans-Golgi network before being secreted into the extracellular space and blood as a hexameric lipoprotein particle [18]. Previous studies demonstrated that NS1 could be involved in vascular leakage and coagulation dysfunction during dengue fever infection through a variety of different mechanisms, including induction of HPA-1 activation by histone L, binding to TLR4 and thrombinogen [19–21]. Apart from its involvement in the pathogenesis of DHF/DSS, NS1 is also involved in regulating early events of viral RNA replication by binding to complement proteins and altering their function, leading to immune escape, etc. [22]. Thereby, NS1 is a multifunctional protein that plays an important role in the pathogenesis of DENV. It was reported that NS1 participates in the regulation of autophagy during DENV infection. NS1 interacts with the autophagy-associated protein Beclin-1 during DENV infection to attenuate Beclin-1 cleavage and thereby promote autophagy to prevent apoptosis and increase viral replication in the early stages of infection [23]. Also, NS1 induced the degradation of autophagy marker p62 and the conversion of LC3-I to LC3-II in HMEC-1 cells, which were involved in the occurrence of autophagy in DENV-infected endothelial cells [24]. It is evident that NS1-induced autophagy contributes to viral replication and thus increases dengue infection. However, whether the mechanism of NS1 involvement in autophagy involves the AMPK/ERK/mTOR signaling pathway has not been studied. In this study, we tentatively determined that NS1 regulates the AMPK/ERK/mTOR signaling pathway most likely among the major nonstructural proteins of DENV-2 with the help of molecular docking analysis, and further experiments confirmed that NS1 induces autophagy via the AMPK/ERK/mTOR signaling pathway. The above results reveal that NS1 is the viral protein that regulates the AMPK/ERK/mTOR signaling pathway to induce autophagy upon DENV-2 infection of HUVEC.

There are three structural domains in NS1 monomer, β-roll, wing and β-ladder. It was found that the protrusions generated by the β-roll and linker structural domains make one side of the NS1 dimer hydrophobic and allow interaction with other transmembrane viral proteins [25]. The Wing structural domain is the major surface-exposed antigenic site of NS1 and a highly conserved region among all four DENV serotypes. Antibodies against the NS1Wing domain peptide protect against DENV infection [26]. Meanwhile, the Wing structural domain is one of the most immunodominant compartments in human and mouse [27]. The WWG motif of the NS1 Wing domain is critical for the initial attachment of NS1 to cells, whereas the β-ladder domain primarily serves in NS1-mediated endothelial dysfunction (21). In the current study, we identified a direct interaction between NS1 and AMPK and co-localization in HUVEC cells. After censoring the different structural domains of NS1, we discovered that the Wing structural domain had the most dramatic effect on the activation of AMPK/ERK/mTOR signaling pathway by NS1 to induce autophagy. It is implied that the Wing structural domain may be a key structural domain for NS1-AMPK interaction, which was probably due to the important role of Wing structural domain in the initial attachment of NS1 to cells (21). Further assays confirmed that the NS1-Wing structural domain interacts with AMPK, illustrating that the NS1-Wing structural domain is a key structural domain in the interaction between NS1 and AMPK.

Separately, we have found that NS1 promotes AMPK phosphorylation and that phosphorylation of Thr172 on the α subunit is the main event required for full AMPK activation, thus selecting the AMPKα subunit for the following study [28]. We discovered that NS1 interacts with the AMPKα subunit. Interestingly, NS1 binds to all three structural domains on the AMPKα subunit, implying that NS1 may promote AMPK phosphorylation through multiple mechanisms. The mechanism could be acting on the kinase structural domain to indirectly promote AMPK activation, or acting on its own inhibitory structural domain to reduce its inhibition of kinase structural domain activation, but the STC and C-terminal structural domains bind more strongly to NS1, so we infer that the kinase structural domain may play a major role. Given that NS1 has no kinase effect, we introduced an upstream kinase that acts on the AMPK kinase structural domain.

LKB1 and CaMKKβ are the main upstream kinases acting on Thr172 phosphorylation in the activation loop of AMPK kinase structural domain in mammals [29, 30]. To determine which kinase is involved in the activation of AMPK by NS1, the preliminary prediction using molecular docking revealed that LKB1 was more likely to bind to NS1, and LKB1 was chosen for the study. Next, NS1 was observed to promote the expression of LKB1, and the knockdown of LKB1 diminished the effect of activation of AMPK/ERK/mTOR pathway by NS1 and NS1-wing structural domains to induce autophagy. Further experiments showed that NS1 interacted with LKB1 and both were mainly localized to the cytosolic membrane. Moreover, the NS1-Wing structural domain also interacts with LKB1, suggesting that NS1 can also bind to LKB1, an upstream kinase of AMPK, which may be a potential mechanism of AMPK activation by NS1. Taken together, NS1 may use its Wing structural domain as a platform for interactions between AMPK and LKB1, which in turn promotes AMPK activation. The data from GST pulldown assay verified the conjecture that in the absence of DENV-2 NS1, AMPKα1, AMPKα2 and LKB1 did not interact with each other, and when HA-NS1 was included in the reaction system, AMPKα1 and LKB1 interacted with each other, but AMPKα2 and LKB1 did not interact with each other. This indicates that NS1 binding to AMPK and LKB1 promoted the interaction of AMPK upstream kinase LKB1 with AMPKα1 to activate AMPK, which in turn activated the AMPK signaling pathway to induce autophagy.

## Conclusion

The present study provides new insights into the mechanism of DENV-induced autophagy and offers some evidence for the experimental design of drugs targeting NS1, which is expected to be a therapeutic target for DENV.

## Materials and methods

### Main regents

The DENV-2 NGC strain was preserved in liquid nitrogen in our laboratory. SV40T-transformed human umbilical vein endothelial cells (PUMC-HUVEC-T1) were provided by Sciencell Research Laboratories. BR-V108 vector, Age I / EcoR I digested; LV-007 vector, Age I and Bam HI digested, were purchased from Shanghai GenePro Technology Co. Ltd. Experimental strain: TOP10 E. coli receptor cells, purchased from TIANGEN. Software: Discovery studio software, MODELLER, Rosettafold and database.

### Virus infection

HUVEC cells were seeded in 12-well plates at a density of 5 × 10^4^ cells/well, and infected with DENV when the cell confluence reached 80%. The cells were then stained according to the condition of MOI = 10, and incubated at 37 °C for 1 h after infection. After incubation, the cells were washed with normal culture medium and placed back into it for continued cultivation.

### Molecular docking

The structures of the target proteins were obtained using MODELLER, Rosettafold and database search, and the predicted proteins were overlaid with the natural proteins and the protein pull-down plots were used to evaluate the protein structures. ZDOCK and RDOCK under the Dock and Analyze Protein Complexes module in Discovery Studio 2019 workbench were used for protein molecular docking and PyMOL was used to assist in mapping analysis.

### Construction of DENV-2 NS1, NS3 and NS5 protein models

The protein sequences of DENV-2 NS1, NS3 and NS5 were obtained from the NCBI database (GenBank: KM204118.1) and saved as fasta files for backup. Perform a protein sequence similarity search in the Protein Data Bank database using PSIBLAST in the NCBI Protein Data Bank. Homologous protein templates were obtained and homology modeling was performed using Modeller 10.1 program. 5 models were generated by the program modeling and the model with the lowest molPDF and DOPE score was selected as the target protein according to the program scoring value. The constructed target protein models were optimized in the GROMACS 2021. program to eliminate the unreasonable structures in the protein models.

The resolved protein structures were obtained in Protein Data Bank, and the target protein models were overlaid with the natural protein structures in Pymol software. The Ramachandran plot of the target protein model was plotted and the stereochemistry of the target protein model was examined using the Chart module in Discovery studio 2019.

### ERK, mTOR, LKB1, CAMKK2 protein structure acquisition

The protein sequences of LKB1, CAMPKK2 and ERK were obtained in the NCBI database with GenbankIDs NM_000455.5, NM_001270485.2 and NM_002745.5, respectively, and the sequence information was saved as.fasta files for backup. The sequence information was submitted to RoseTTAFold for ab initio protein structure prediction to obtain the structural model of the target protein. Search in Protein Data Bank to mine the crystal structure information of mTOR protein, screen to identify PDB ID: 4JSV as the source of mTOR protein structure, download the.PDB file of 4JSV, delete other proteins and small molecule structures in Pymol, save the separate mTOR protein structure for backup.

### Protein docking ZDOCK calculation

The proteins DENV-2 NS1, NS3, and NS5 were subjected to ZDOCK calculations with AMPKα1 subunit, and DENV-2 NS1 was subjected to ZDOCK calculations with ERK, mTOR, LKB1, and CAMKK2 proteins, respectively, in seven groups, one for each group. The same set of two protein structures were placed in the same window and calculated using Dock Protei (ZDOCK) in Macromolecular|Dock and Analyze Protein Complexes, and AMPKα1, ERK, mTOR, LKB1 and CAMKK2 were selected as receptors, respectively, and DENV-2 NS1, NS3 and NS5 are ligands, and the parameters Angular is 6, RMSD Cutoff is 10, Interface Cuoff is 10, Maximum Number of Clusters is 100, and other parameters are defaulted for ZDOCK calculation.

### Protein docking RDOCK calculation

The screened Pose was endowed with CHARMm force field and calculated using Refine Docked proteins (RDOCK) in Macromolecular|Dock and Analyze Protein Complexes, Input Receptor Protein selected InputPoses select Create New Group From Selection, other parameters are default, and the screened proteins are used as a new group. The proteins after screening are used as a new group for RDOCK calculation.

### Co-IP assay

Cells were collected and lysed in RIPA lysis buffer (Beyotime) containing protease inhibitor cocktail (Roche Diagnostics). Whole cell lysates (2 mg) were precleared with 30 μL protein G beads (Life Technologies), followed by the addition of 2 μg isotype-matched IgG control or the indicated antibody and incubated for 2 h on a shaking platform. Immunoprecipitates were collected by centrifugation and then resolved by SDS-PAGE.

### GST pulldown assay

The pGEX-2T was used as a vector, and the Gateway cassette was inserted after the GST sequence using Overlap PCR, and the target gene was inserted along the ORF by Gateway technology to construct an expression vector for the GST-tagged protein. ArcticExpress E. coli was used as the host bacterium and IPTG was used as the inducer to induce GST-tagged protein and fusion protein expression at 15 ℃. The purified GST fusion protein beads and HA-tagged proteins (5–10% reserved as input) were mixed at 4 °C for 12 h. Add anti-HA or anti-GST beads and incubate with fusion protein for another 4 h. These small beads were washed three times and then the proteins were detected by protein blotting.

### Immunofluorescence assay

The treated samples were fixed with paraformaldehyde for 10 min. The cells were then permeabilized with 2% Triton X-100 (Solarbio) for 10 min. Samples were washed three times with PBS. After blocking the cells with 5% BSA (Solarbio) g for 30 min, the cells were incubated with primary antibodies (anti-[1:250 anti-AMPK, 1:200 anti-NS1], fluorescent secondary antibodies) for 1 h at 37C. Finally, cells were incubated with 2-(4-amidinophenyl)-1H-indole-6-carboxamidine (DAPI, Solarbio) for 10 min at 37C and then observed under a laser confocal microscope.

### Western blotting

Cells from each treatment group were collected and fully lysed by adding cell lysis solution (PMSF:RIPA = 1: 100) and protease and phosphoprotease inhibitors. The extracted proteins were subjected to SDS-PAGE electrophoresis and then transferred to polyvinylidene fluoride (PVDF) membranes. The PVDF membranes were blocked for 2 h and then incubated with the primary antibody overnight at 4 degrees Celsius. After three washes with Tris-buffered saline-Tween-20 (TBST) (Additional file 1), the membrane was incubated with the secondary antibody for l h at 37 °C, followed by three washes with TBST. The ECL luminescent substrate was added to the PVDF membrane and color development was performed. The density of immunoreactive protein bands was analyzed using Quantity One software (Bio-Rad, Hercules, CA).

### Statistical methods

The experimental data were analyzed using the statistical analysis software SPSS 25.0 (Statistical Product and Service Solutions 25.0), all experiments had more than three replications, and the data were plotted using GraphPad Prism 8.0 software. The measurement data conforming to normal distribution were expressed as mean ± standard deviation (${\overline{\text{x}}}$ ± s), and one-way ANOVA was used for comparison between groups, and t-test was used for two-way comparison, and *P* < 0.05 indicated that the difference was statistically significant.

### Supplementary Information

**Additional file 1.** Original images of Western blotting.

## Data Availability

The datasets used or analysed during the current study are available from the corresponding author on reasonable request.
