# Supplementary figures and images for: DENV-2 NS1 promotes AMPK-LKB1 interaction to activate AMPK/ERK/mTOR signaling pathway to induce autophagy (part 2 of 2)
Source: Virol J. 2023 Oct 11;20:231. doi: 10.1186/s12985-023-02166-0 (PMC10568820; doi:10.1186/s12985-023-02166-0)

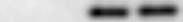

Supplement: Supplementary file 1 — Additional file 1. Original images of Western blotting. [file 12985_2023_2166_MOESM1_ESM.zip › supplementary file/Fig.7/B/IP NS1-wd/Input/NS1-mut.png]

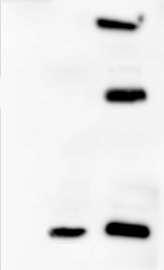

Supplement: Supplementary file 1 — Additional file 1. Original images of Western blotting. [file 12985_2023_2166_MOESM1_ESM.zip › supplementary file/Fig.7/C/原始图片/GST-NS1.jpg]

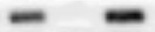

Supplement: Supplementary file 1 — Additional file 1. Original images of Western blotting. [file 12985_2023_2166_MOESM1_ESM.zip › supplementary file/Fig.7/C/原始图片/LKB1.jpg]

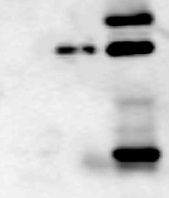

Supplement: Supplementary file 1 — Additional file 1. Original images of Western blotting. [file 12985_2023_2166_MOESM1_ESM.zip › supplementary file/Fig.7/D/原始图/GST-NS1-wd.jpg]

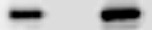

Supplement: Supplementary file 1 — Additional file 1. Original images of Western blotting. [file 12985_2023_2166_MOESM1_ESM.zip › supplementary file/Fig.7/D/原始图/LKB1.jpg]

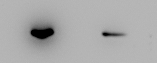

Supplement: Supplementary file 1 — Additional file 1. Original images of Western blotting. [file 12985_2023_2166_MOESM1_ESM.zip › supplementary file/Fig.7/E/原始图片/AK2..tif]

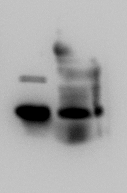

Supplement: Supplementary file 1 — Additional file 1. Original images of Western blotting. [file 12985_2023_2166_MOESM1_ESM.zip › supplementary file/Fig.7/E/原始图片/AK2_.tif]

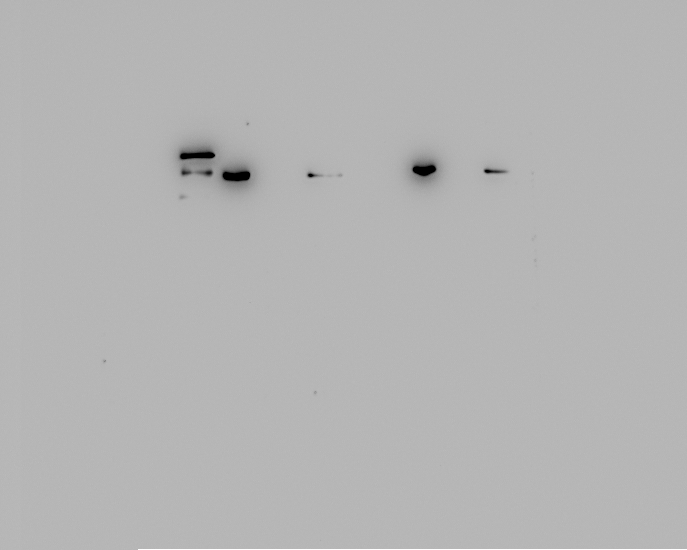

Supplement: Supplementary file 1 — Additional file 1. Original images of Western blotting. [file 12985_2023_2166_MOESM1_ESM.zip › supplementary file/Fig.7/E/完整膜/AK2..tif]

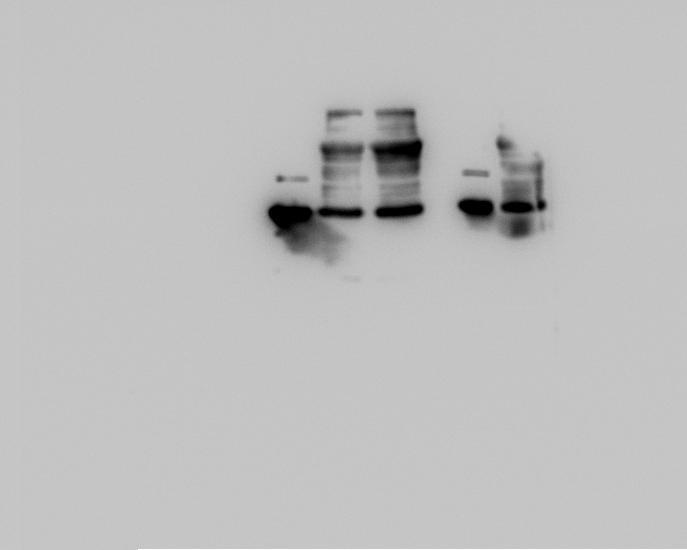

Supplement: Supplementary file 1 — Additional file 1. Original images of Western blotting. [file 12985_2023_2166_MOESM1_ESM.zip › supplementary file/Fig.7/E/完整膜/AK2_.tif]

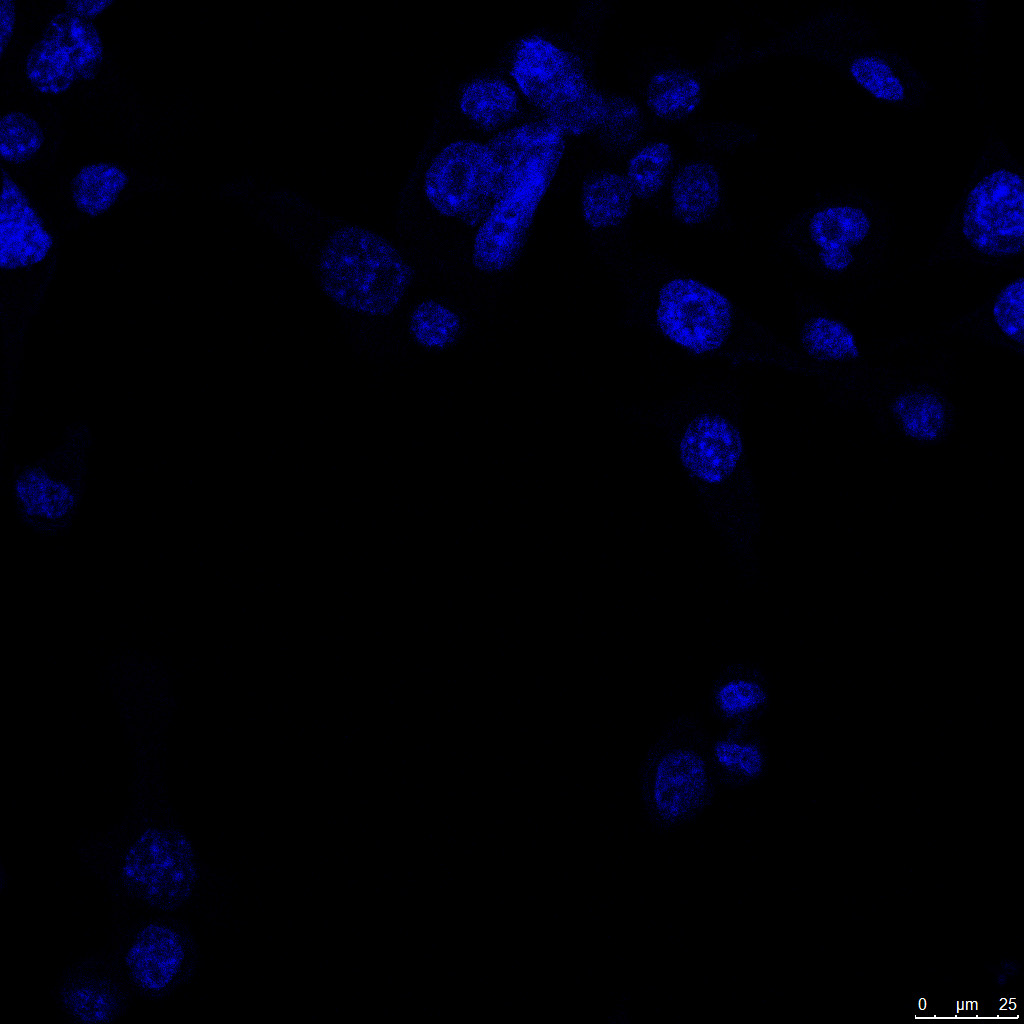

Supplement: Supplementary file 1 — Additional file 1. Original images of Western blotting. [file 12985_2023_2166_MOESM1_ESM.zip › supplementary file/Fig.7/F/DAPI.tif]

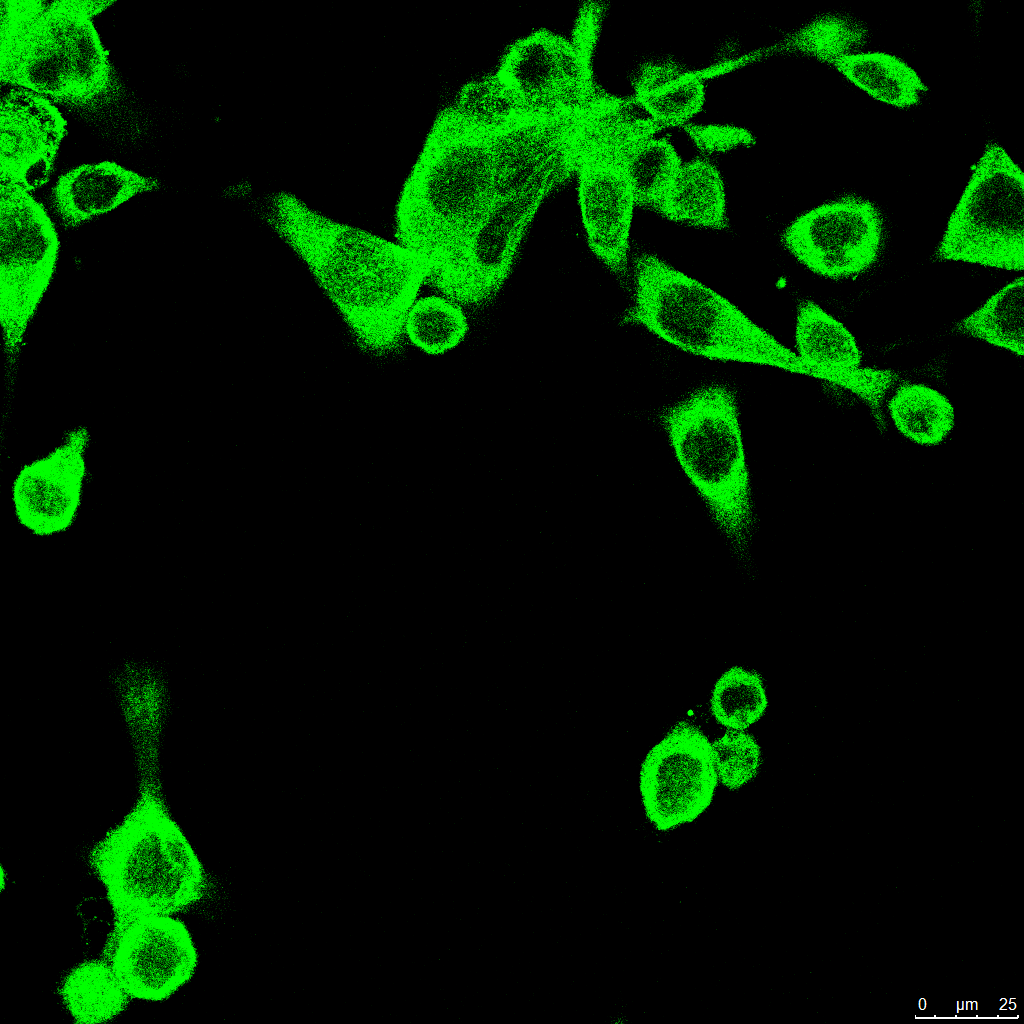

Supplement: Supplementary file 1 — Additional file 1. Original images of Western blotting. [file 12985_2023_2166_MOESM1_ESM.zip › supplementary file/Fig.7/F/LKB1.tif]

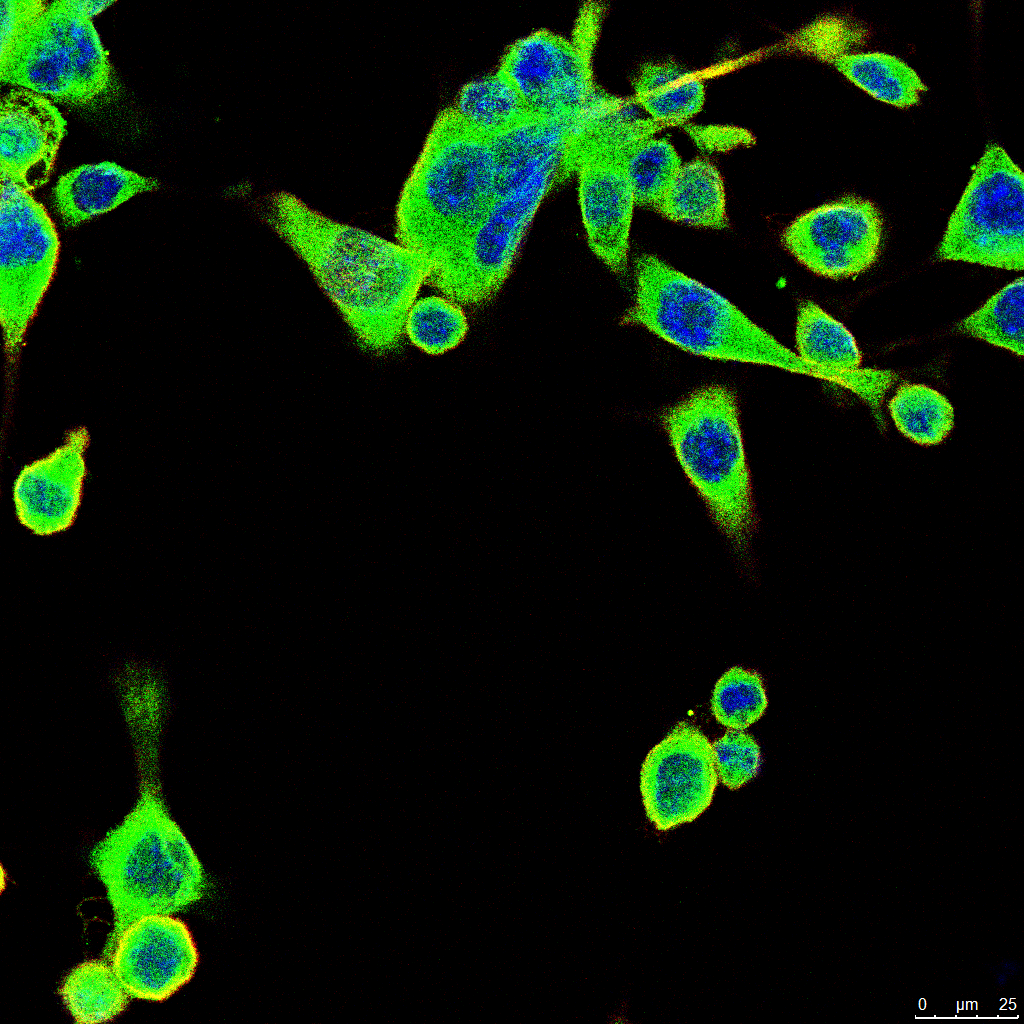

Supplement: Supplementary file 1 — Additional file 1. Original images of Western blotting. [file 12985_2023_2166_MOESM1_ESM.zip › supplementary file/Fig.7/F/Merge.tif]

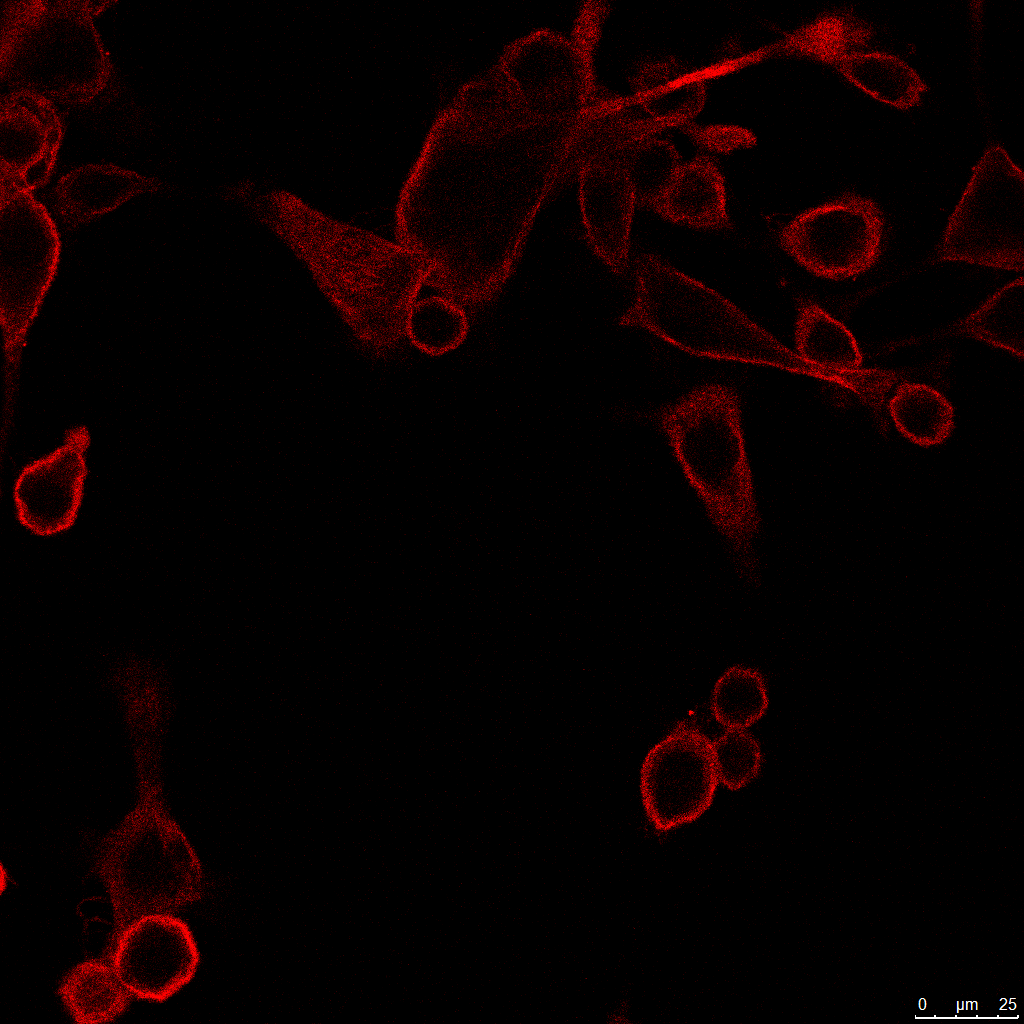

Supplement: Supplementary file 1 — Additional file 1. Original images of Western blotting. [file 12985_2023_2166_MOESM1_ESM.zip › supplementary file/Fig.7/F/NS1.tif]

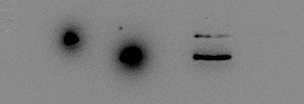

Supplement: Supplementary file 1 — Additional file 1. Original images of Western blotting. [file 12985_2023_2166_MOESM1_ESM.zip › supplementary file/Fig.7/G/原始图片/AK1 .tif]

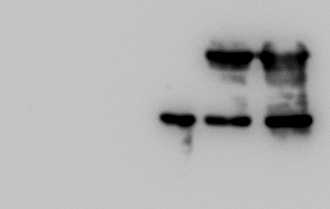

Supplement: Supplementary file 1 — Additional file 1. Original images of Western blotting. [file 12985_2023_2166_MOESM1_ESM.zip › supplementary file/Fig.7/G/原始图片/AK1.tif]

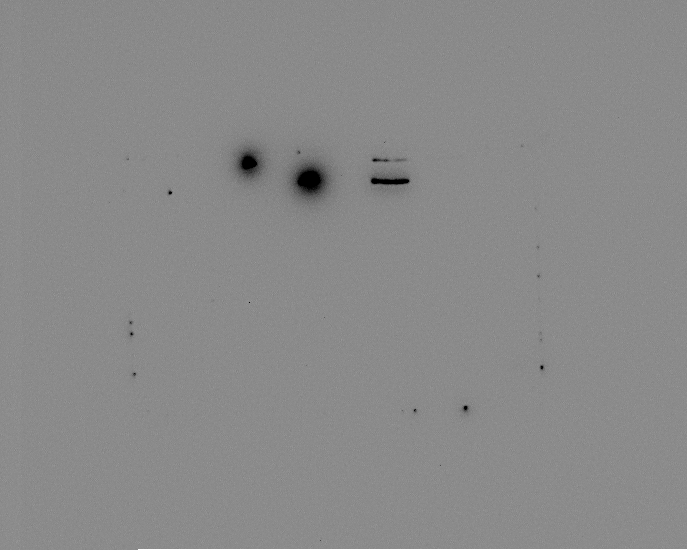

Supplement: Supplementary file 1 — Additional file 1. Original images of Western blotting. [file 12985_2023_2166_MOESM1_ESM.zip › supplementary file/Fig.7/G/完整膜/AK1 .tif]

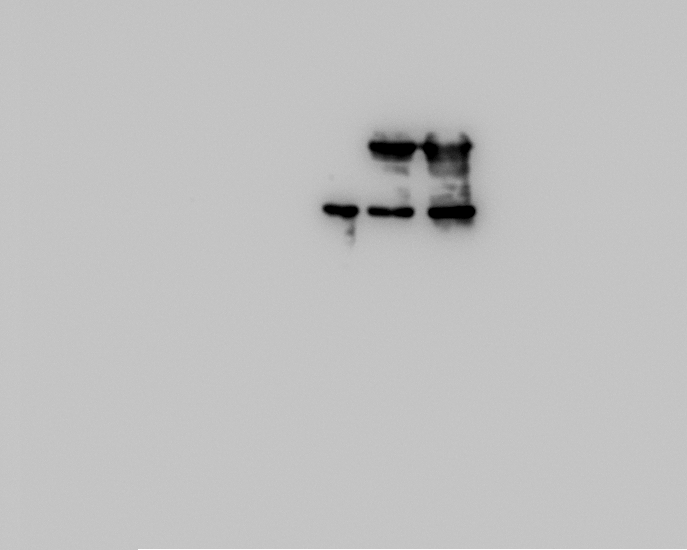

Supplement: Supplementary file 1 — Additional file 1. Original images of Western blotting. [file 12985_2023_2166_MOESM1_ESM.zip › supplementary file/Fig.7/G/完整膜/AK1.tif]

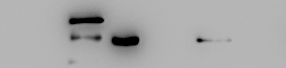

Supplement: Supplementary file 1 — Additional file 1. Original images of Western blotting. [file 12985_2023_2166_MOESM1_ESM.zip › supplementary file/Fig.7/H/原始图片/AK2..tif]

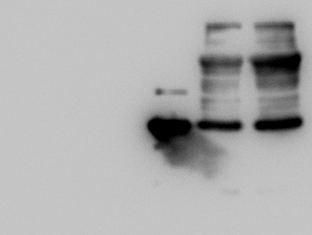

Supplement: Supplementary file 1 — Additional file 1. Original images of Western blotting. [file 12985_2023_2166_MOESM1_ESM.zip › supplementary file/Fig.7/H/原始图片/AK2_.tif]
