# Supplementary figures and images for: DENV-2 NS1 promotes AMPK-LKB1 interaction to activate AMPK/ERK/mTOR signaling pathway to induce autophagy (part 1 of 2)
Source: Virol J. 2023 Oct 11;20:231. doi: 10.1186/s12985-023-02166-0 (PMC10568820; doi:10.1186/s12985-023-02166-0)

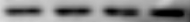

Supplement: Supplementary file 1 — Additional file 1. Original images of Western blotting. [file 12985_2023_2166_MOESM1_ESM.zip › supplementary file/Fig.2A/AMPK.png]

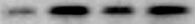

Supplement: Supplementary file 1 — Additional file 1. Original images of Western blotting. [file 12985_2023_2166_MOESM1_ESM.zip › supplementary file/Fig.2A/beclin1.png]

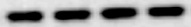

Supplement: Supplementary file 1 — Additional file 1. Original images of Western blotting. [file 12985_2023_2166_MOESM1_ESM.zip › supplementary file/Fig.2A/ERK.png]

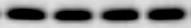

Supplement: Supplementary file 1 — Additional file 1. Original images of Western blotting. [file 12985_2023_2166_MOESM1_ESM.zip › supplementary file/Fig.2A/GAPDH.png]

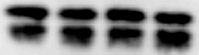

Supplement: Supplementary file 1 — Additional file 1. Original images of Western blotting. [file 12985_2023_2166_MOESM1_ESM.zip › supplementary file/Fig.2A/LC3B.png]

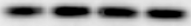

Supplement: Supplementary file 1 — Additional file 1. Original images of Western blotting. [file 12985_2023_2166_MOESM1_ESM.zip › supplementary file/Fig.2A/mTOR.png]

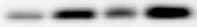

Supplement: Supplementary file 1 — Additional file 1. Original images of Western blotting. [file 12985_2023_2166_MOESM1_ESM.zip › supplementary file/Fig.2A/NS1.png]

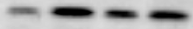

Supplement: Supplementary file 1 — Additional file 1. Original images of Western blotting. [file 12985_2023_2166_MOESM1_ESM.zip › supplementary file/Fig.2A/p-AMPK.png]

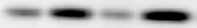

Supplement: Supplementary file 1 — Additional file 1. Original images of Western blotting. [file 12985_2023_2166_MOESM1_ESM.zip › supplementary file/Fig.2A/p-ERK.png]

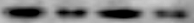

Supplement: Supplementary file 1 — Additional file 1. Original images of Western blotting. [file 12985_2023_2166_MOESM1_ESM.zip › supplementary file/Fig.2A/p-mTOR.png]

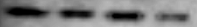

Supplement: Supplementary file 1 — Additional file 1. Original images of Western blotting. [file 12985_2023_2166_MOESM1_ESM.zip › supplementary file/Fig.2A/p62.png]

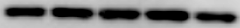

Supplement: Supplementary file 1 — Additional file 1. Original images of Western blotting. [file 12985_2023_2166_MOESM1_ESM.zip › supplementary file/Fig.2B/AMPK.png]

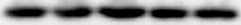

Supplement: Supplementary file 1 — Additional file 1. Original images of Western blotting. [file 12985_2023_2166_MOESM1_ESM.zip › supplementary file/Fig.2B/beclin1.png]

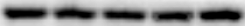

Supplement: Supplementary file 1 — Additional file 1. Original images of Western blotting. [file 12985_2023_2166_MOESM1_ESM.zip › supplementary file/Fig.2B/ERK.png]

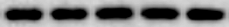

Supplement: Supplementary file 1 — Additional file 1. Original images of Western blotting. [file 12985_2023_2166_MOESM1_ESM.zip › supplementary file/Fig.2B/GAPDH.png]

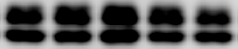

Supplement: Supplementary file 1 — Additional file 1. Original images of Western blotting. [file 12985_2023_2166_MOESM1_ESM.zip › supplementary file/Fig.2B/LC3B.png]

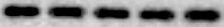

Supplement: Supplementary file 1 — Additional file 1. Original images of Western blotting. [file 12985_2023_2166_MOESM1_ESM.zip › supplementary file/Fig.2B/mTOR.png]

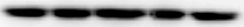

Supplement: Supplementary file 1 — Additional file 1. Original images of Western blotting. [file 12985_2023_2166_MOESM1_ESM.zip › supplementary file/Fig.2B/p-AMPK.png]

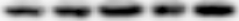

Supplement: Supplementary file 1 — Additional file 1. Original images of Western blotting. [file 12985_2023_2166_MOESM1_ESM.zip › supplementary file/Fig.2B/p-ERK.png]

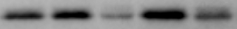

Supplement: Supplementary file 1 — Additional file 1. Original images of Western blotting. [file 12985_2023_2166_MOESM1_ESM.zip › supplementary file/Fig.2B/p-mTOR.png]

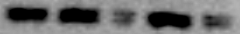

Supplement: Supplementary file 1 — Additional file 1. Original images of Western blotting. [file 12985_2023_2166_MOESM1_ESM.zip › supplementary file/Fig.2B/P62.png]

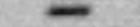

Supplement: Supplementary file 1 — Additional file 1. Original images of Western blotting. [file 12985_2023_2166_MOESM1_ESM.zip › supplementary file/Fig.3A/IP NS1/IB/AMPK.png]

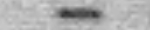

Supplement: Supplementary file 1 — Additional file 1. Original images of Western blotting. [file 12985_2023_2166_MOESM1_ESM.zip › supplementary file/Fig.3A/IP NS1/IB/NS1.png]

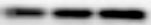

Supplement: Supplementary file 1 — Additional file 1. Original images of Western blotting. [file 12985_2023_2166_MOESM1_ESM.zip › supplementary file/Fig.3A/IP NS1/Input/AMPK.png]

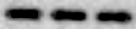

Supplement: Supplementary file 1 — Additional file 1. Original images of Western blotting. [file 12985_2023_2166_MOESM1_ESM.zip › supplementary file/Fig.3A/IP NS1/Input/GAPDH.png]

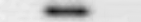

Supplement: Supplementary file 1 — Additional file 1. Original images of Western blotting. [file 12985_2023_2166_MOESM1_ESM.zip › supplementary file/Fig.3A/IP NS1/Input/NS1.png]

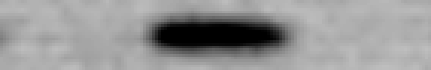

Supplement: Supplementary file 1 — Additional file 1. Original images of Western blotting. [file 12985_2023_2166_MOESM1_ESM.zip › supplementary file/Fig.3B/IB/AMPK.png]

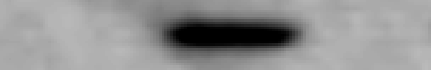

Supplement: Supplementary file 1 — Additional file 1. Original images of Western blotting. [file 12985_2023_2166_MOESM1_ESM.zip › supplementary file/Fig.3B/IB/NS1.png]

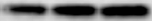

Supplement: Supplementary file 1 — Additional file 1. Original images of Western blotting. [file 12985_2023_2166_MOESM1_ESM.zip › supplementary file/Fig.3B/Input/AMPK.tif]

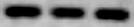

Supplement: Supplementary file 1 — Additional file 1. Original images of Western blotting. [file 12985_2023_2166_MOESM1_ESM.zip › supplementary file/Fig.3B/Input/GAPDH.tif]

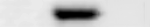

Supplement: Supplementary file 1 — Additional file 1. Original images of Western blotting. [file 12985_2023_2166_MOESM1_ESM.zip › supplementary file/Fig.3B/Input/NS1.tif]

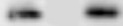

Supplement: Supplementary file 1 — Additional file 1. Original images of Western blotting. [file 12985_2023_2166_MOESM1_ESM.zip › supplementary file/Fig.3C/AMPK2.jpg]

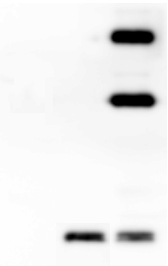

Supplement: Supplementary file 1 — Additional file 1. Original images of Western blotting. [file 12985_2023_2166_MOESM1_ESM.zip › supplementary file/Fig.3C/GST-NS1.png]

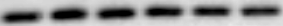

Supplement: Supplementary file 1 — Additional file 1. Original images of Western blotting. [file 12985_2023_2166_MOESM1_ESM.zip › supplementary file/Fig.4A/AMPK.png]

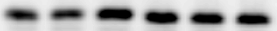

Supplement: Supplementary file 1 — Additional file 1. Original images of Western blotting. [file 12985_2023_2166_MOESM1_ESM.zip › supplementary file/Fig.4A/beclin1.png]

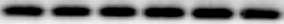

Supplement: Supplementary file 1 — Additional file 1. Original images of Western blotting. [file 12985_2023_2166_MOESM1_ESM.zip › supplementary file/Fig.4A/ERK.png]

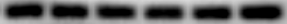

Supplement: Supplementary file 1 — Additional file 1. Original images of Western blotting. [file 12985_2023_2166_MOESM1_ESM.zip › supplementary file/Fig.4A/GAPDH.png]

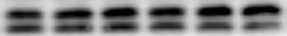

Supplement: Supplementary file 1 — Additional file 1. Original images of Western blotting. [file 12985_2023_2166_MOESM1_ESM.zip › supplementary file/Fig.4A/LC3B.png]

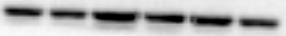

Supplement: Supplementary file 1 — Additional file 1. Original images of Western blotting. [file 12985_2023_2166_MOESM1_ESM.zip › supplementary file/Fig.4A/LKB1.png]

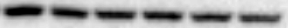

Supplement: Supplementary file 1 — Additional file 1. Original images of Western blotting. [file 12985_2023_2166_MOESM1_ESM.zip › supplementary file/Fig.4A/mTOR.png]

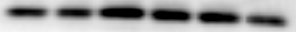

Supplement: Supplementary file 1 — Additional file 1. Original images of Western blotting. [file 12985_2023_2166_MOESM1_ESM.zip › supplementary file/Fig.4A/NS1-OE.png]

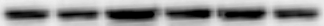

Supplement: Supplementary file 1 — Additional file 1. Original images of Western blotting. [file 12985_2023_2166_MOESM1_ESM.zip › supplementary file/Fig.4A/p-AMPK.png]

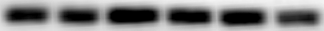

Supplement: Supplementary file 1 — Additional file 1. Original images of Western blotting. [file 12985_2023_2166_MOESM1_ESM.zip › supplementary file/Fig.4A/p-ERK.png]

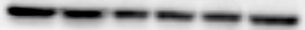

Supplement: Supplementary file 1 — Additional file 1. Original images of Western blotting. [file 12985_2023_2166_MOESM1_ESM.zip › supplementary file/Fig.4A/p-mTOR.png]

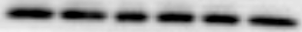

Supplement: Supplementary file 1 — Additional file 1. Original images of Western blotting. [file 12985_2023_2166_MOESM1_ESM.zip › supplementary file/Fig.4A/P62.png]

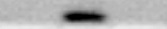

Supplement: Supplementary file 1 — Additional file 1. Original images of Western blotting. [file 12985_2023_2166_MOESM1_ESM.zip › supplementary file/Fig.4B/IB/NS1.jpg]

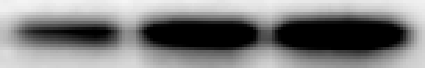

Supplement: Supplementary file 1 — Additional file 1. Original images of Western blotting. [file 12985_2023_2166_MOESM1_ESM.zip › supplementary file/Fig.4B/input/AMPK.png]

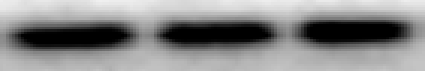

Supplement: Supplementary file 1 — Additional file 1. Original images of Western blotting. [file 12985_2023_2166_MOESM1_ESM.zip › supplementary file/Fig.4B/input/GAPDH.png]

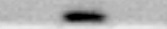

Supplement: Supplementary file 1 — Additional file 1. Original images of Western blotting. [file 12985_2023_2166_MOESM1_ESM.zip › supplementary file/Fig.4B/input/NS1.png]

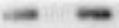

Supplement: Supplementary file 1 — Additional file 1. Original images of Western blotting. [file 12985_2023_2166_MOESM1_ESM.zip › supplementary file/Fig.4C/AMPK.png]

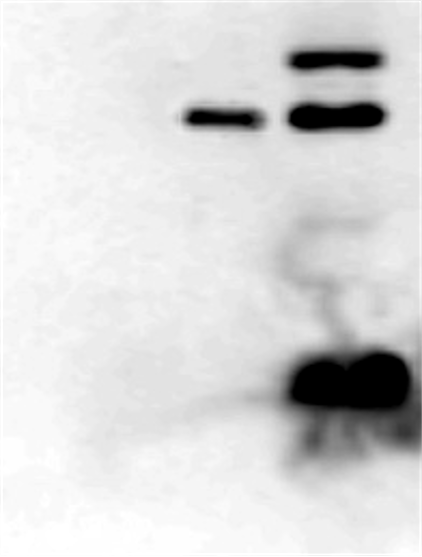

Supplement: Supplementary file 1 — Additional file 1. Original images of Western blotting. [file 12985_2023_2166_MOESM1_ESM.zip › supplementary file/Fig.4C/wing domain.png]

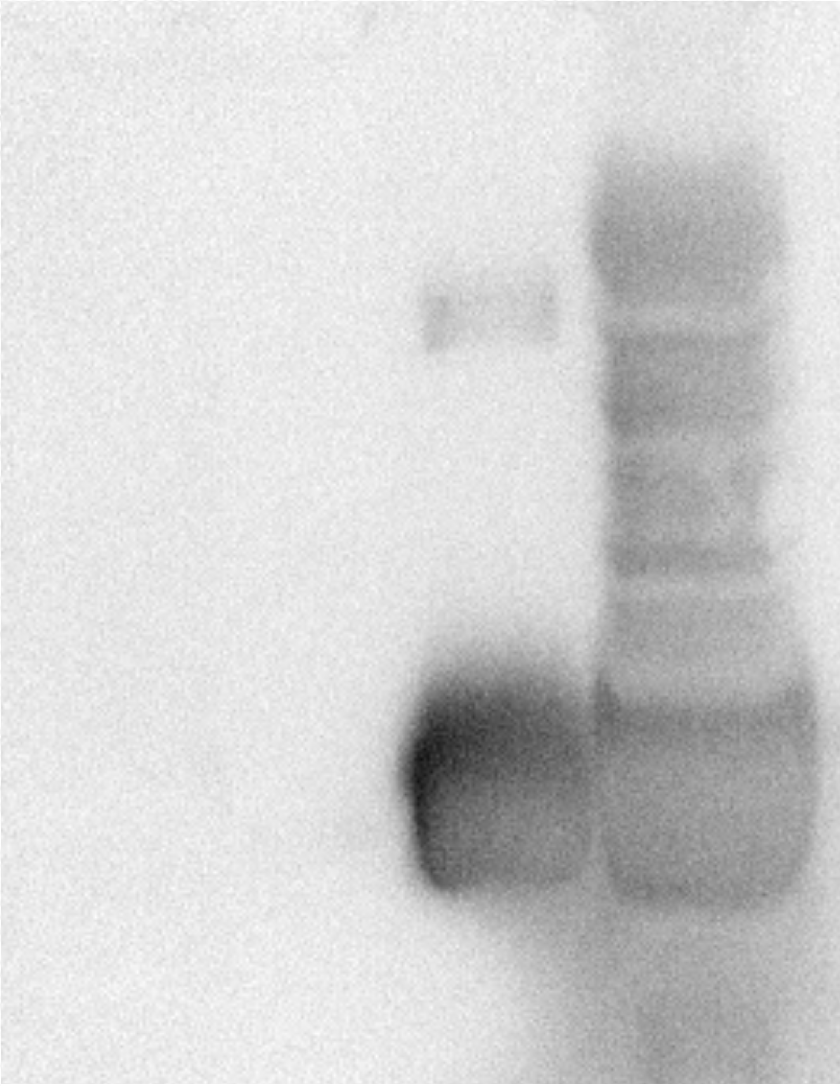

Supplement: Supplementary file 1 — Additional file 1. Original images of Western blotting. [file 12985_2023_2166_MOESM1_ESM.zip › supplementary file/Fig.5/A/GST-NS1.tif]

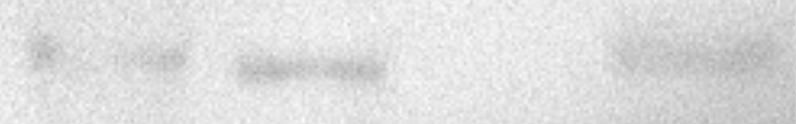

Supplement: Supplementary file 1 — Additional file 1. Original images of Western blotting. [file 12985_2023_2166_MOESM1_ESM.zip › supplementary file/Fig.5/A/HA-AMPKα1.tif]

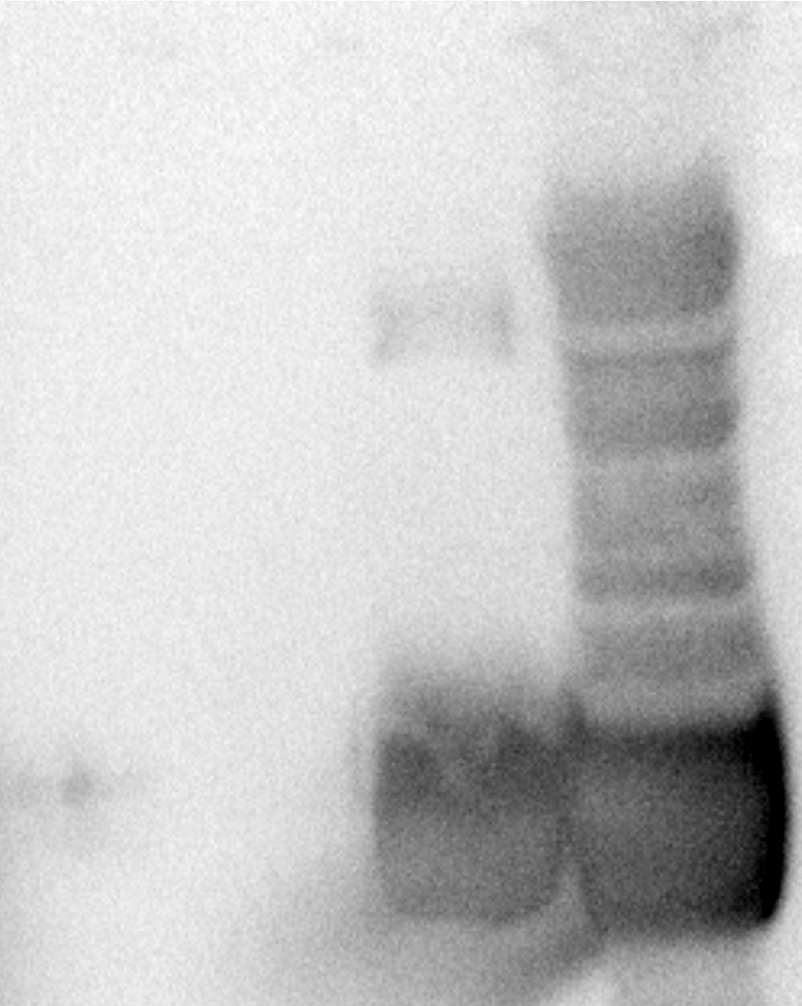

Supplement: Supplementary file 1 — Additional file 1. Original images of Western blotting. [file 12985_2023_2166_MOESM1_ESM.zip › supplementary file/Fig.5/B/GST-NS1.tif]

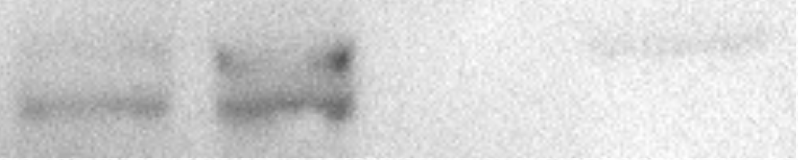

Supplement: Supplementary file 1 — Additional file 1. Original images of Western blotting. [file 12985_2023_2166_MOESM1_ESM.zip › supplementary file/Fig.5/B/HA-AMPKα2.tif]

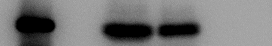

Supplement: Supplementary file 1 — Additional file 1. Original images of Western blotting. [file 12985_2023_2166_MOESM1_ESM.zip › supplementary file/Fig.5/C/原始条带/AK1C .tif]

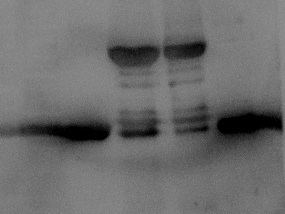

Supplement: Supplementary file 1 — Additional file 1. Original images of Western blotting. [file 12985_2023_2166_MOESM1_ESM.zip › supplementary file/Fig.5/C/原始条带/AK1C.tif]

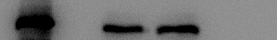

Supplement: Supplementary file 1 — Additional file 1. Original images of Western blotting. [file 12985_2023_2166_MOESM1_ESM.zip › supplementary file/Fig.5/C/原始条带/AK1S .tif]

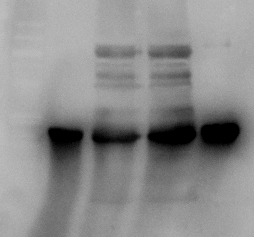

Supplement: Supplementary file 1 — Additional file 1. Original images of Western blotting. [file 12985_2023_2166_MOESM1_ESM.zip › supplementary file/Fig.5/C/原始条带/AK1S .tif]

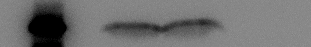

Supplement: Supplementary file 1 — Additional file 1. Original images of Western blotting. [file 12985_2023_2166_MOESM1_ESM.zip › supplementary file/Fig.5/C/原始条带/AK1U .tif]

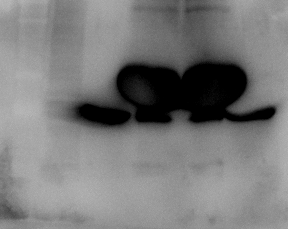

Supplement: Supplementary file 1 — Additional file 1. Original images of Western blotting. [file 12985_2023_2166_MOESM1_ESM.zip › supplementary file/Fig.5/C/原始条带/AK1U.tif]

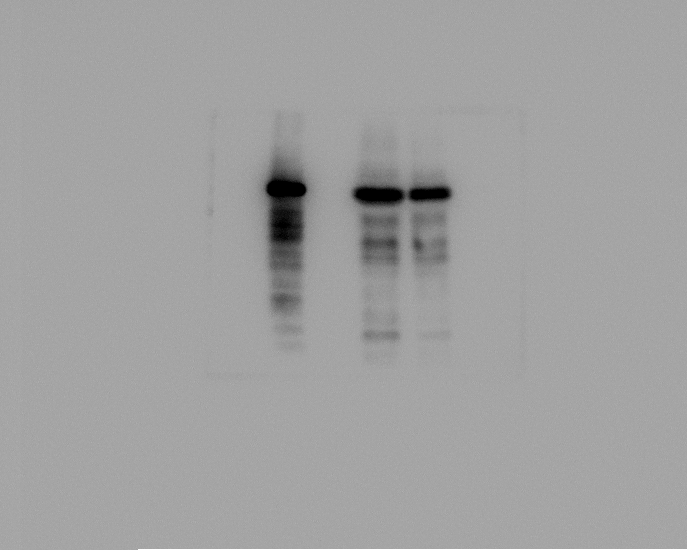

Supplement: Supplementary file 1 — Additional file 1. Original images of Western blotting. [file 12985_2023_2166_MOESM1_ESM.zip › supplementary file/Fig.5/C/完整膜/AK1C .tif]

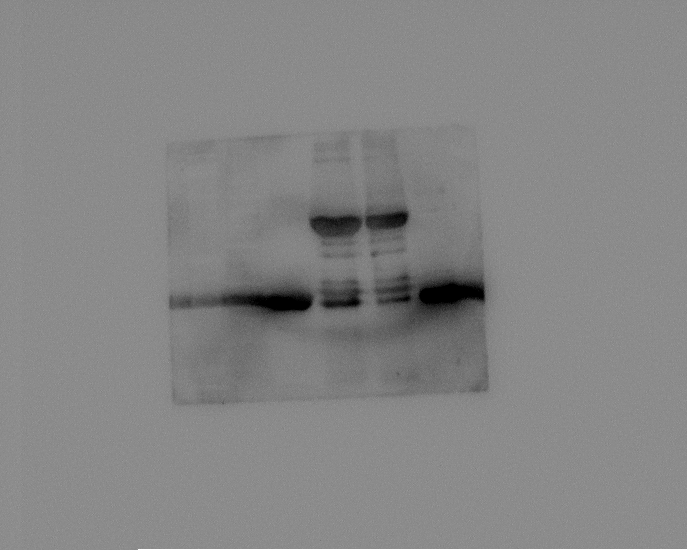

Supplement: Supplementary file 1 — Additional file 1. Original images of Western blotting. [file 12985_2023_2166_MOESM1_ESM.zip › supplementary file/Fig.5/C/完整膜/AK1C.tif]

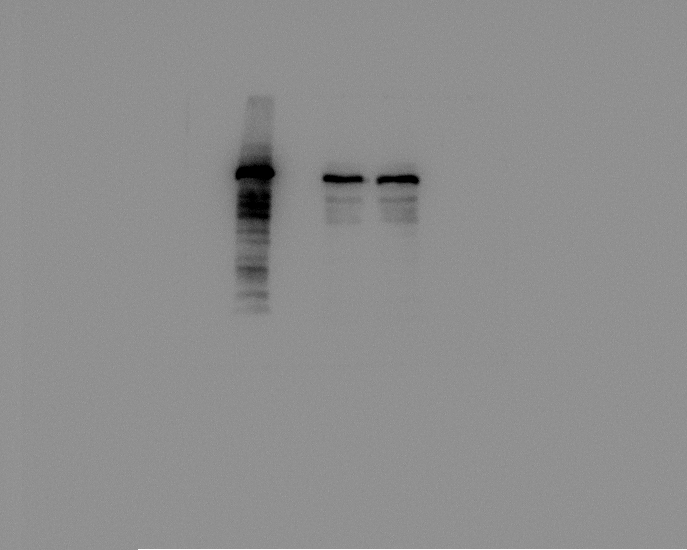

Supplement: Supplementary file 1 — Additional file 1. Original images of Western blotting. [file 12985_2023_2166_MOESM1_ESM.zip › supplementary file/Fig.5/C/完整膜/AK1S .tif]

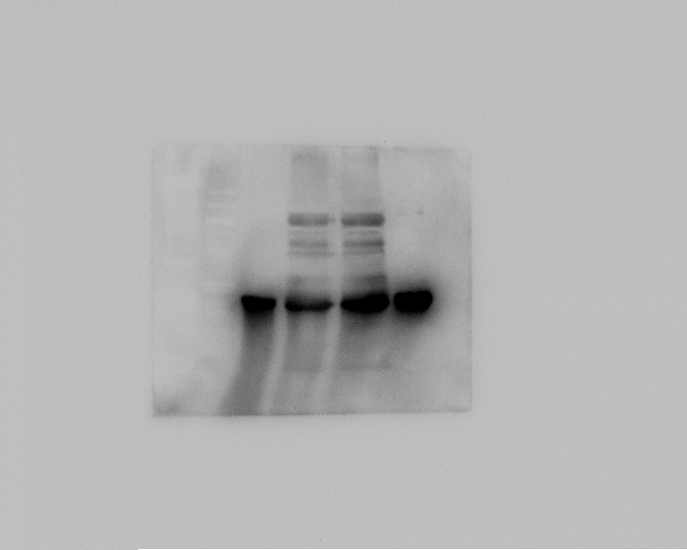

Supplement: Supplementary file 1 — Additional file 1. Original images of Western blotting. [file 12985_2023_2166_MOESM1_ESM.zip › supplementary file/Fig.5/C/完整膜/AK1S .tif]

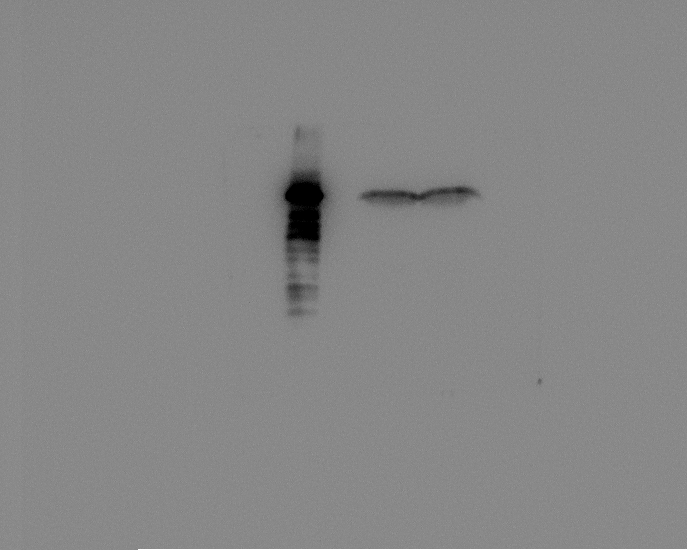

Supplement: Supplementary file 1 — Additional file 1. Original images of Western blotting. [file 12985_2023_2166_MOESM1_ESM.zip › supplementary file/Fig.5/C/完整膜/AK1U .tif]

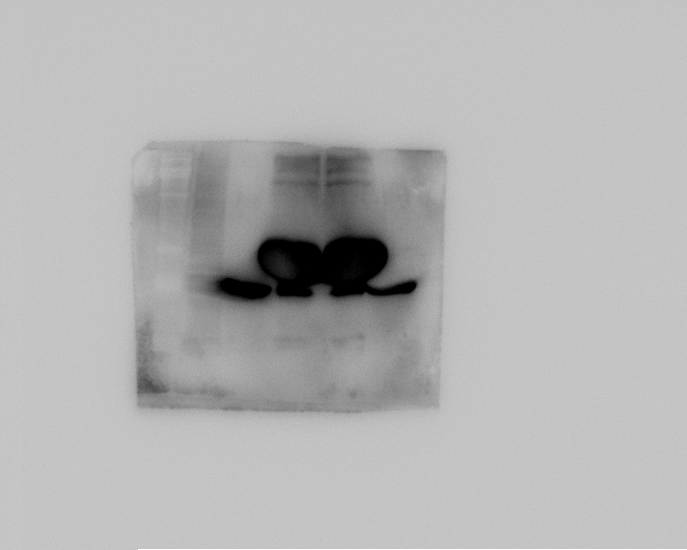

Supplement: Supplementary file 1 — Additional file 1. Original images of Western blotting. [file 12985_2023_2166_MOESM1_ESM.zip › supplementary file/Fig.5/C/完整膜/AK1U.tif]

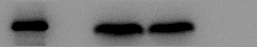

Supplement: Supplementary file 1 — Additional file 1. Original images of Western blotting. [file 12985_2023_2166_MOESM1_ESM.zip › supplementary file/Fig.5/D/原始图片/AK2C .tif]

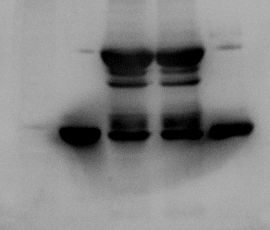

Supplement: Supplementary file 1 — Additional file 1. Original images of Western blotting. [file 12985_2023_2166_MOESM1_ESM.zip › supplementary file/Fig.5/D/原始图片/AK2C.tif]

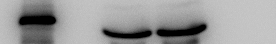

Supplement: Supplementary file 1 — Additional file 1. Original images of Western blotting. [file 12985_2023_2166_MOESM1_ESM.zip › supplementary file/Fig.5/D/原始图片/AK2S .tif]

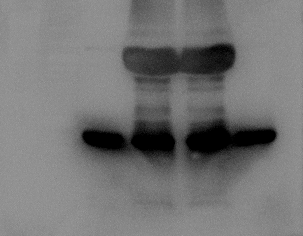

Supplement: Supplementary file 1 — Additional file 1. Original images of Western blotting. [file 12985_2023_2166_MOESM1_ESM.zip › supplementary file/Fig.5/D/原始图片/AK2S.tif]

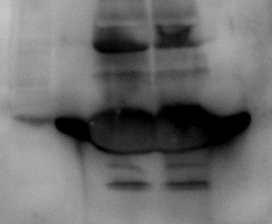

Supplement: Supplementary file 1 — Additional file 1. Original images of Western blotting. [file 12985_2023_2166_MOESM1_ESM.zip › supplementary file/Fig.5/D/原始图片/AK2U .tif]

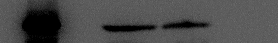

Supplement: Supplementary file 1 — Additional file 1. Original images of Western blotting. [file 12985_2023_2166_MOESM1_ESM.zip › supplementary file/Fig.5/D/原始图片/AK2U.tif]

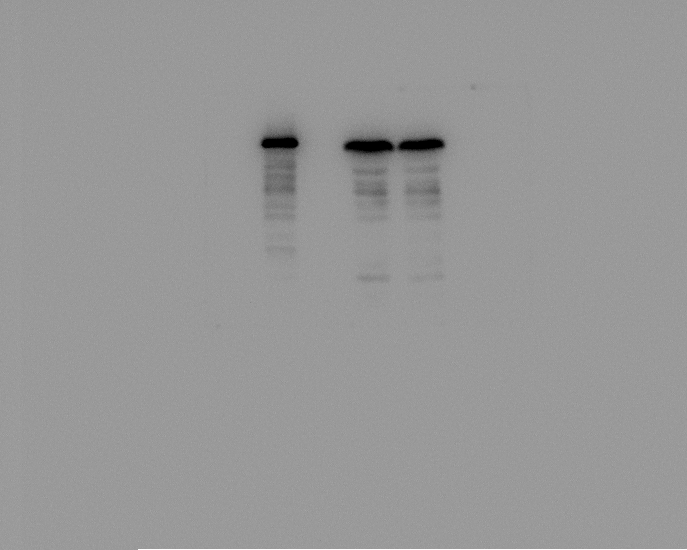

Supplement: Supplementary file 1 — Additional file 1. Original images of Western blotting. [file 12985_2023_2166_MOESM1_ESM.zip › supplementary file/Fig.5/D/原始完整图片/AK2C .tif]

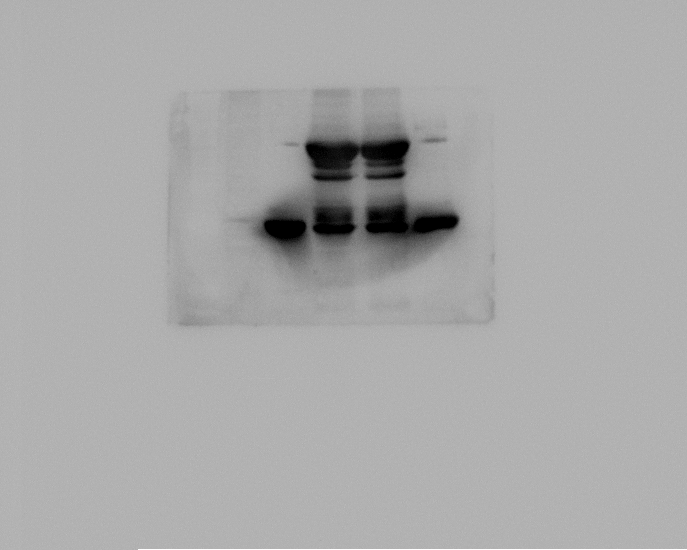

Supplement: Supplementary file 1 — Additional file 1. Original images of Western blotting. [file 12985_2023_2166_MOESM1_ESM.zip › supplementary file/Fig.5/D/原始完整图片/AK2C.tif]

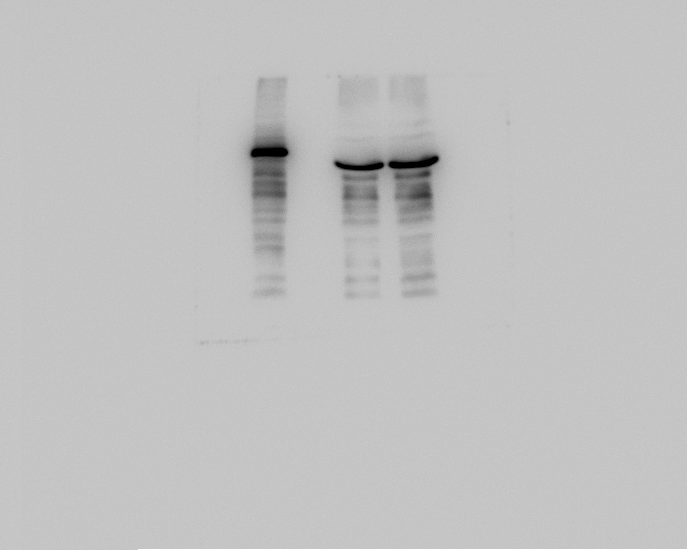

Supplement: Supplementary file 1 — Additional file 1. Original images of Western blotting. [file 12985_2023_2166_MOESM1_ESM.zip › supplementary file/Fig.5/D/原始完整图片/AK2S .tif]

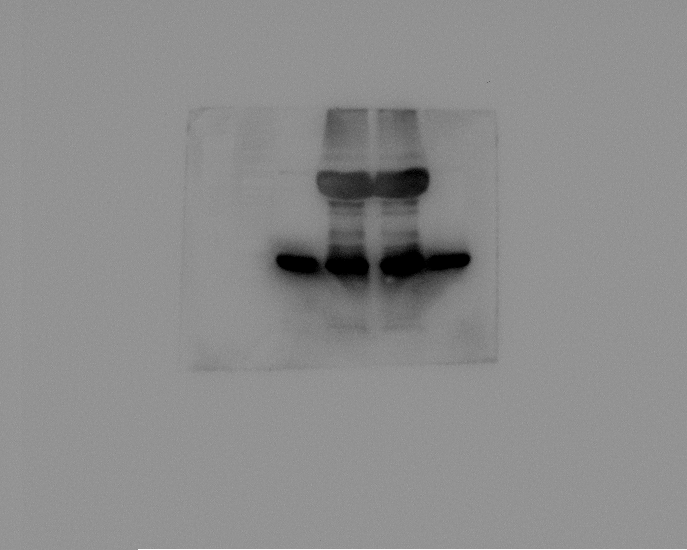

Supplement: Supplementary file 1 — Additional file 1. Original images of Western blotting. [file 12985_2023_2166_MOESM1_ESM.zip › supplementary file/Fig.5/D/原始完整图片/AK2S.tif]

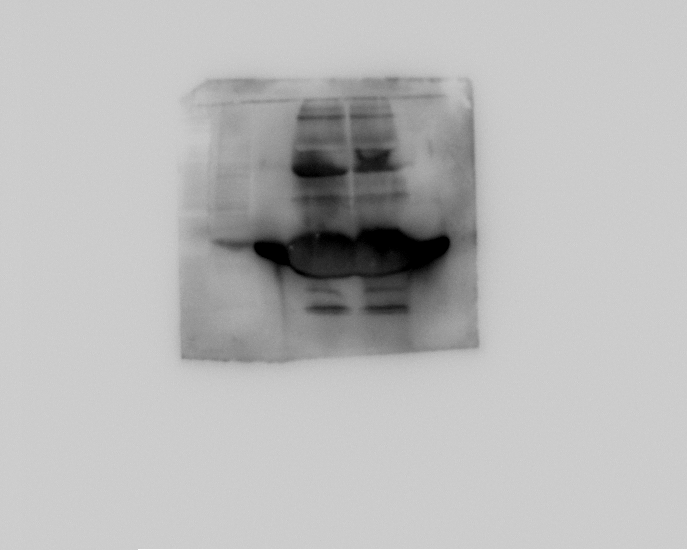

Supplement: Supplementary file 1 — Additional file 1. Original images of Western blotting. [file 12985_2023_2166_MOESM1_ESM.zip › supplementary file/Fig.5/D/原始完整图片/AK2U .tif]

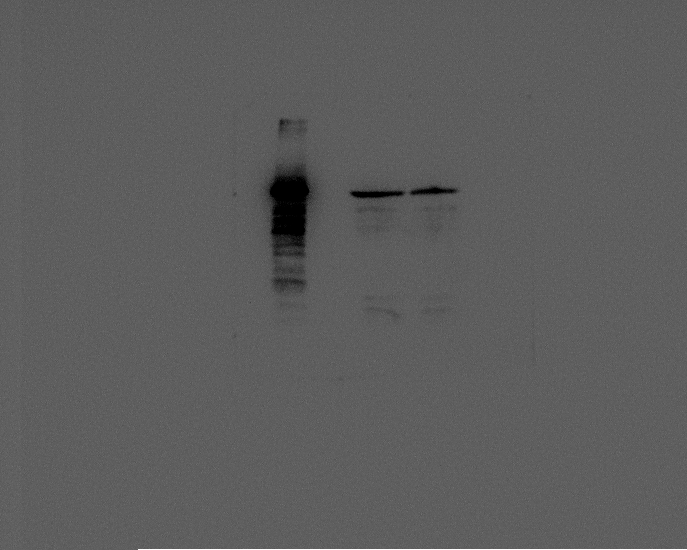

Supplement: Supplementary file 1 — Additional file 1. Original images of Western blotting. [file 12985_2023_2166_MOESM1_ESM.zip › supplementary file/Fig.5/D/原始完整图片/AK2U.tif]

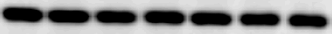

Supplement: Supplementary file 1 — Additional file 1. Original images of Western blotting. [file 12985_2023_2166_MOESM1_ESM.zip › supplementary file/Fig.6B/原始图片/GAPDH.png]

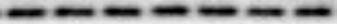

Supplement: Supplementary file 1 — Additional file 1. Original images of Western blotting. [file 12985_2023_2166_MOESM1_ESM.zip › supplementary file/Fig.6C/AMPK.png]

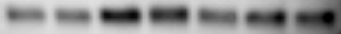

Supplement: Supplementary file 1 — Additional file 1. Original images of Western blotting. [file 12985_2023_2166_MOESM1_ESM.zip › supplementary file/Fig.6C/Beclin1.png]

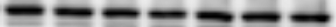

Supplement: Supplementary file 1 — Additional file 1. Original images of Western blotting. [file 12985_2023_2166_MOESM1_ESM.zip › supplementary file/Fig.6C/ERK.png]

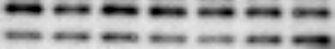

Supplement: Supplementary file 1 — Additional file 1. Original images of Western blotting. [file 12985_2023_2166_MOESM1_ESM.zip › supplementary file/Fig.6C/LC3B.png]

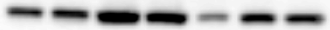

Supplement: Supplementary file 1 — Additional file 1. Original images of Western blotting. [file 12985_2023_2166_MOESM1_ESM.zip › supplementary file/Fig.6C/LKB1.png]

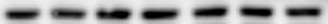

Supplement: Supplementary file 1 — Additional file 1. Original images of Western blotting. [file 12985_2023_2166_MOESM1_ESM.zip › supplementary file/Fig.6C/mTOR.png]

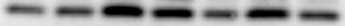

Supplement: Supplementary file 1 — Additional file 1. Original images of Western blotting. [file 12985_2023_2166_MOESM1_ESM.zip › supplementary file/Fig.6C/NS1-OE.png]

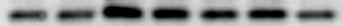

Supplement: Supplementary file 1 — Additional file 1. Original images of Western blotting. [file 12985_2023_2166_MOESM1_ESM.zip › supplementary file/Fig.6C/p-AMPK.png]

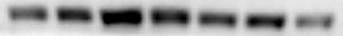

Supplement: Supplementary file 1 — Additional file 1. Original images of Western blotting. [file 12985_2023_2166_MOESM1_ESM.zip › supplementary file/Fig.6C/p-ERK.png]

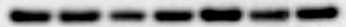

Supplement: Supplementary file 1 — Additional file 1. Original images of Western blotting. [file 12985_2023_2166_MOESM1_ESM.zip › supplementary file/Fig.6C/p-mTOR.png]

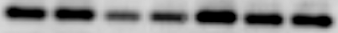

Supplement: Supplementary file 1 — Additional file 1. Original images of Western blotting. [file 12985_2023_2166_MOESM1_ESM.zip › supplementary file/Fig.6C/p62.png]

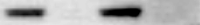

Supplement: Supplementary file 1 — Additional file 1. Original images of Western blotting. [file 12985_2023_2166_MOESM1_ESM.zip › supplementary file/Fig.7/A/IP NS1/IB/LKB1.png]

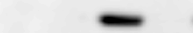

Supplement: Supplementary file 1 — Additional file 1. Original images of Western blotting. [file 12985_2023_2166_MOESM1_ESM.zip › supplementary file/Fig.7/A/IP NS1/IB/NS1.png]

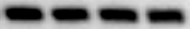

Supplement: Supplementary file 1 — Additional file 1. Original images of Western blotting. [file 12985_2023_2166_MOESM1_ESM.zip › supplementary file/Fig.7/A/IP NS1/Input/GAPDH.png]

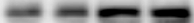

Supplement: Supplementary file 1 — Additional file 1. Original images of Western blotting. [file 12985_2023_2166_MOESM1_ESM.zip › supplementary file/Fig.7/A/IP NS1/Input/LKB1.png]

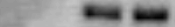

Supplement: Supplementary file 1 — Additional file 1. Original images of Western blotting. [file 12985_2023_2166_MOESM1_ESM.zip › supplementary file/Fig.7/A/IP NS1/Input/NS1.png]

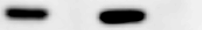

Supplement: Supplementary file 1 — Additional file 1. Original images of Western blotting. [file 12985_2023_2166_MOESM1_ESM.zip › supplementary file/Fig.7/B/IP NS1-wd/IB/LKB1.png]

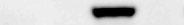

Supplement: Supplementary file 1 — Additional file 1. Original images of Western blotting. [file 12985_2023_2166_MOESM1_ESM.zip › supplementary file/Fig.7/B/IP NS1-wd/IB/NS1-mut.png]

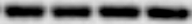

Supplement: Supplementary file 1 — Additional file 1. Original images of Western blotting. [file 12985_2023_2166_MOESM1_ESM.zip › supplementary file/Fig.7/B/IP NS1-wd/Input/GAPDH.png]

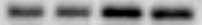

Supplement: Supplementary file 1 — Additional file 1. Original images of Western blotting. [file 12985_2023_2166_MOESM1_ESM.zip › supplementary file/Fig.7/B/IP NS1-wd/Input/LKB1.png]
